# Low-cost and automated phenotyping system “Phenomenon” for multi-sensor in situ monitoring in plant in vitro culture

**DOI:** 10.1186/s13007-023-01018-w

**Published:** 2023-05-02

**Authors:** Hans Bethge, Traud Winkelmann, Patrick Lüdeke, Thomas Rath

**Affiliations:** 1 Laboratory for Biosystems Engineering, Faculty of Agricultural Sciences and Landscape Architecture, Osnabrück University of Applied Sciences, Oldenburger Landstraße 24, 49090 Osnabrück, Germany; 2 Institute of Horticultural Production Systems, Section of Woody Plant and Propagation Physiology, Leibniz Universität Hannover, Herrenhäuser Str. 2, 30419 Hannover, Germany; 3 Hannover, Germany

**Keywords:** Chlorophyll fluorescence, Image analysis, Laser distance sensor, Non-destructive growth analysis, Plant tissue culture, RGB imaging, Spectrometer, Thermal sensor

## Abstract

**Background:**

The current development of sensor technologies towards ever more cost-effective and powerful systems is steadily increasing the application of low-cost sensors in different horticultural sectors. In plant in vitro culture, as a fundamental technique for plant breeding and plant propagation, the majority of evaluation methods to describe the performance of these cultures are based on destructive approaches, limiting data to unique endpoint measurements. Therefore, a non-destructive phenotyping system capable of automated, continuous and objective quantification of in vitro plant traits is desirable.

**Results:**

An automated low-cost multi-sensor system acquiring phenotypic data of plant in vitro cultures was developed and evaluated. Unique hardware and software components were selected to construct a xyz-scanning system with an adequate accuracy for consistent data acquisition. Relevant plant growth predictors, such as projected area of explants and average canopy height were determined employing multi-sensory imaging and various developmental processes could be monitored and documented. The validation of the RGB image segmentation pipeline using a random forest classifier revealed very strong correlation with manual pixel annotation. Depth imaging by a laser distance sensor of plant in vitro cultures enabled the description of the dynamic behavior of the average canopy height, the maximum plant height, but also the culture media height and volume. Projected plant area in depth data by RANSAC (random sample consensus) segmentation approach well matched the projected plant area by RGB image processing pipeline. In addition, a successful proof of concept for in situ spectral fluorescence monitoring was achieved and challenges of thermal imaging were documented. Potential use cases for the digital quantification of key performance parameters in research and commercial application are discussed.

**Conclusion:**

The technical realization of “Phenomenon” allows phenotyping of plant in vitro cultures under highly challenging conditions and enables multi-sensory monitoring through closed vessels, ensuring the aseptic status of the cultures. Automated sensor application in plant tissue culture promises great potential for a non-destructive growth analysis enhancing commercial propagation as well as enabling research with novel digital parameters recorded over time.

**Supplementary Information:**

The online version contains supplementary material available at 10.1186/s13007-023-01018-w.

## Background

A bottleneck of the promising discipline “phenomics”, which combines high-throughput phenotyping with genome and transcriptome analyses, is the automated acquisition of phenotypic data [1]. Applications of digital phenotyping range from monitoring individual plant cells in controlled environments to satellite-based remote sensing at the plant canopy level using various ground-based and mobile platforms such as gantries, agricultural vehicles, drones, and various sensor technologies such as LIDAR, RGB camera and spectral devices [1]. Although plant in vitro culture is the basis of most biotechnological methods for breeding and propagation of disease-free plants, very limited research using automated sensors in plant tissue culture has been reported, mainly using “plant to sensor” approaches [2-7] and thus involved a significant degree of invasiveness. So far, only few sensor technologies were used, including monochromatic imaging sensors [2], RGB cameras [3, 5-7], modified RGB camera setups with a near infrared (NIR) channel [4, 8] and thermal imaging sensors [9]. Therefore, most studies (reviewed by Gupta and Karmakar [5]) focused on image analysis to estimate parameters like biomass of callus [10], classification of somatic embryos and regenerated shoots [11, 12], as well as chlorophyll determination [13] and growth of embryogenic suspension cultures [14]. A fully automated image acquisition customized for in vitro cultured plantlets was demonstrated by Dhondt et al. [4]. The “in vitro growth imaging system” (IGIS) consisted of a rotating metal platform (carousel) to capture top-down images of *A. thaliana* rosettes cultivated in Petri dishes. However practical usage of the setup is limited in terms of scalability and it is not suited for phenotyping of cultures of commercially important micropropagated species like *Phalaenopsis* spp., *Rubus* spp. and *Helleborus* spp. [15] due to their larger explant size and height.

Visual monitoring of the cultures is a costly and time-consuming repetitive task [8]—typically once a week in research and depending on the plant species every 2 to 10 weeks in commercial propagation—to assess the plant quality, the occurrence of contaminations, the outgrowth of endophytes, and morphophysiological disorders in research laboratories and commercial micropropagation laboratories. Quantitative assessments, such as biomass increase or multiplication rate, are up to now limited to single point measurements at the end of a subculture. Automation offers great potential for increasing efficiency of micropropagation laboratories since 60–70% of total costs of a micropropagated explant is due to manual labor [16]. According to Cardoso et al. [17], the high cost of labor for skilled workers is the most common reason for plant tissue laboratories to switch from manual to automated processes. However, the switch is currently often hindered by the high initial cost of automation, which increases the interest in low-cost monitoring systems for commercial use.

**Fig. 1:**
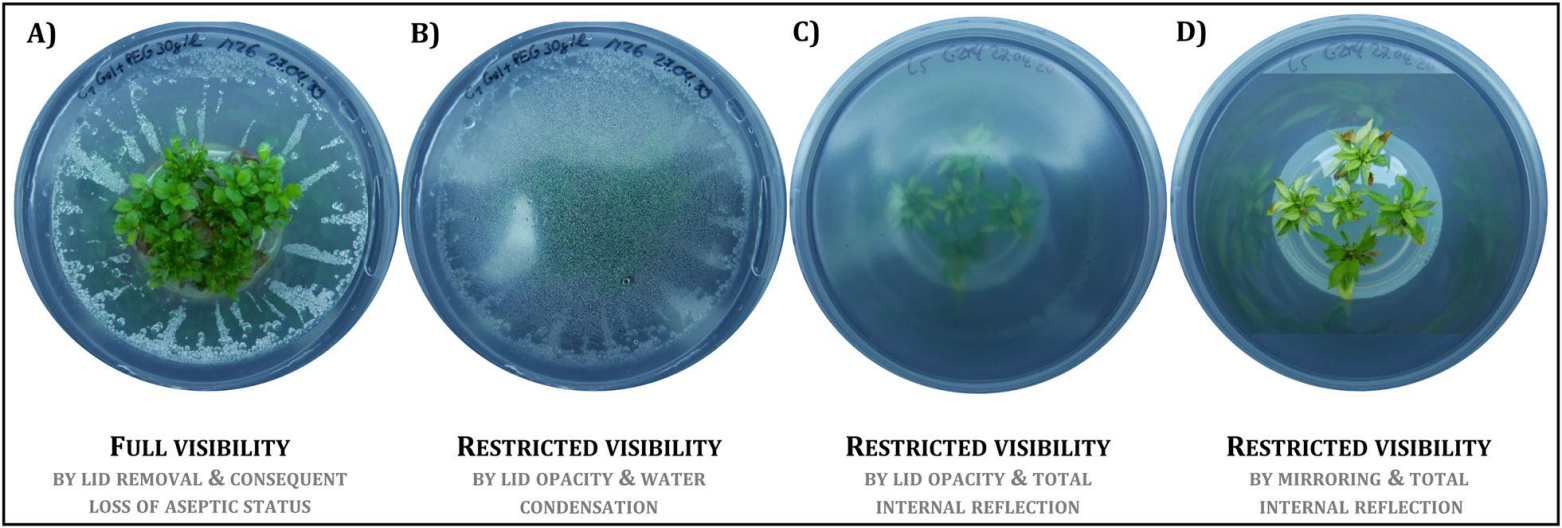
Highly challenging imaging situation of plant in vitro cultures. **A** Culture vessel lid removal offers a proper imaging, but results in the loss of aseptic status of the cultures. Problems for optical monitoring arise from **B** water condensation, **C** opacity of culture containers and total internal reflection of ambient or detection light **D** as well as mirroring of plantlets inside the culture vessel. RGB images were taken from two shoot cultures of *Malus* spp. The image pairs **A**–**B** and **C**–**D** each show one image scene **B**, **C** with and **A**, **D** without lid, respectively

Due to the specific in vitro culture conditions in closed vessels, optical monitoring approaches face a number of challenges such as water condensation on the lid, opacity and total reflection of plastic lids or media surfaces (Fig. 1) [4, 6, 8]. Therefore, most plant evaluation methods were destructive and non-real-time methods, while digital phenotyping of in vitro plants allows objective and continuous quantification of plant characteristics over time. Important biological key parameters for the performance of micropropagated plants include biomass, multiplication rate, shoot length, plant quality, and the absence of malformations, contaminations, and outgrowing endophytes.

Here, we describe the development of a low-cost phenotyping platform (named “Phenomenon”) suitable for direct monitoring of plant in vitro cultures while cultivation in an established multi-layered shelf system. In addition, the “Phenomenon” system is scalable for high-throughput use in commercial laboratories and capable of monitoring a wide range of plant species and various different in vitro culture techniques. In the present study, we aimed (i) to describe in detail the hard- and software components of the established phenotyping system, (ii) to validate the four sensor systems and (iii) to demonstrate the performance of the system for the quantification of growth parameters, such as projected plant area, average canopy and maximum plant height.

## Results

### Phenotyping system concept

**Fig. 2:**
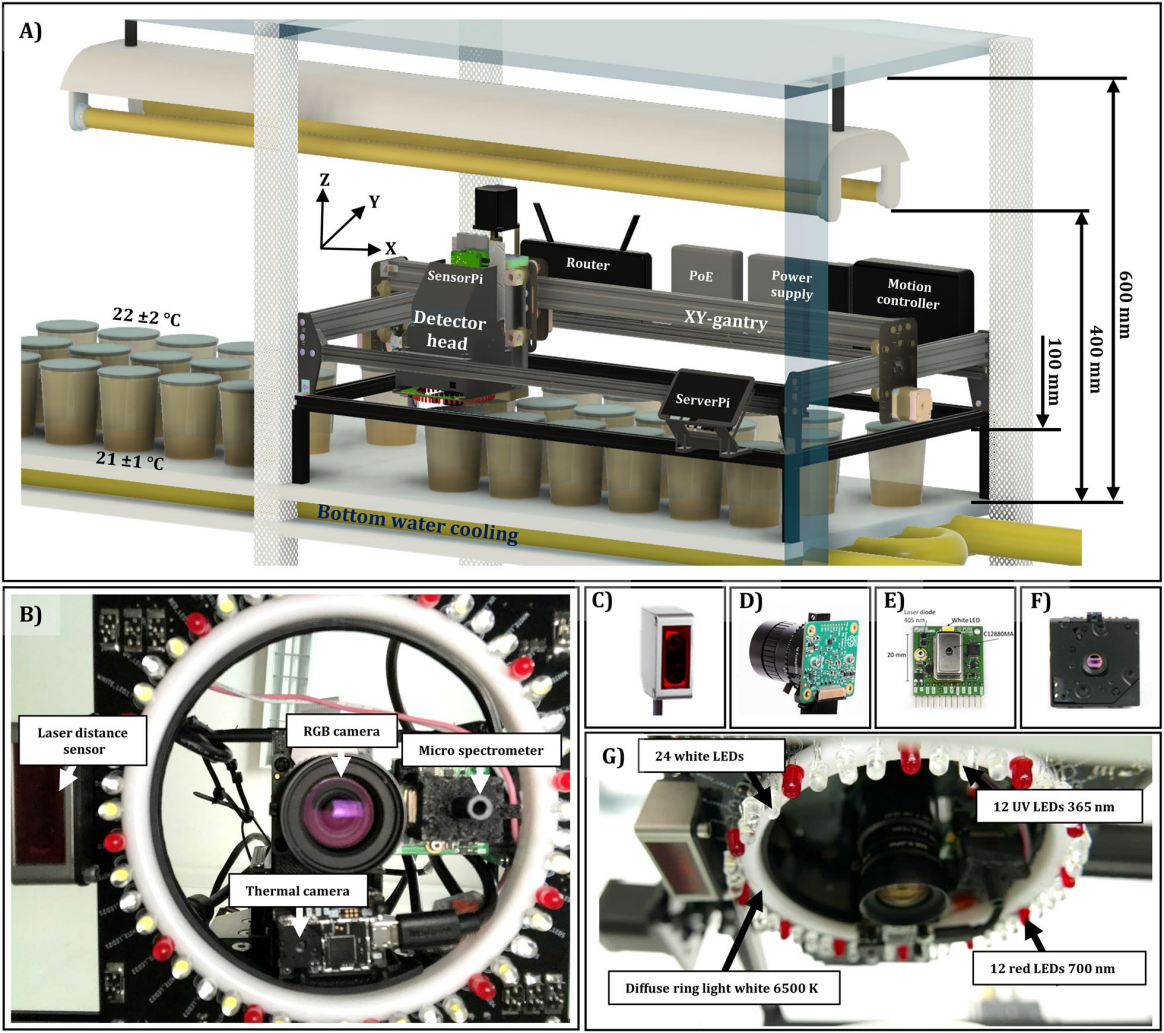
Experimental setup of the phenotyping system designed for direct monitoring of plantlets and explants cultured in vitro. **A** 3D representation of the designed robot platform inside a multi-layered shelf system with bottom water cooling. **B** Closeup of sensor arrangement of the developed multi-sensor detector head. Four different sensors, including **C** a laser distance sensor, **D** RGB camera, **E** a micro spectrometer and **F** a thermal camera defined the multi-sensor detector head. Furthermore, **G** a ring-light printed circuit board, including UV, white and red LEDs was added to a purchasable diffuse ring light to meet the highly specific illumination situation of monitoring plant in vitro cultures. Detailed description in “Methods” section

**Fig. 3:**
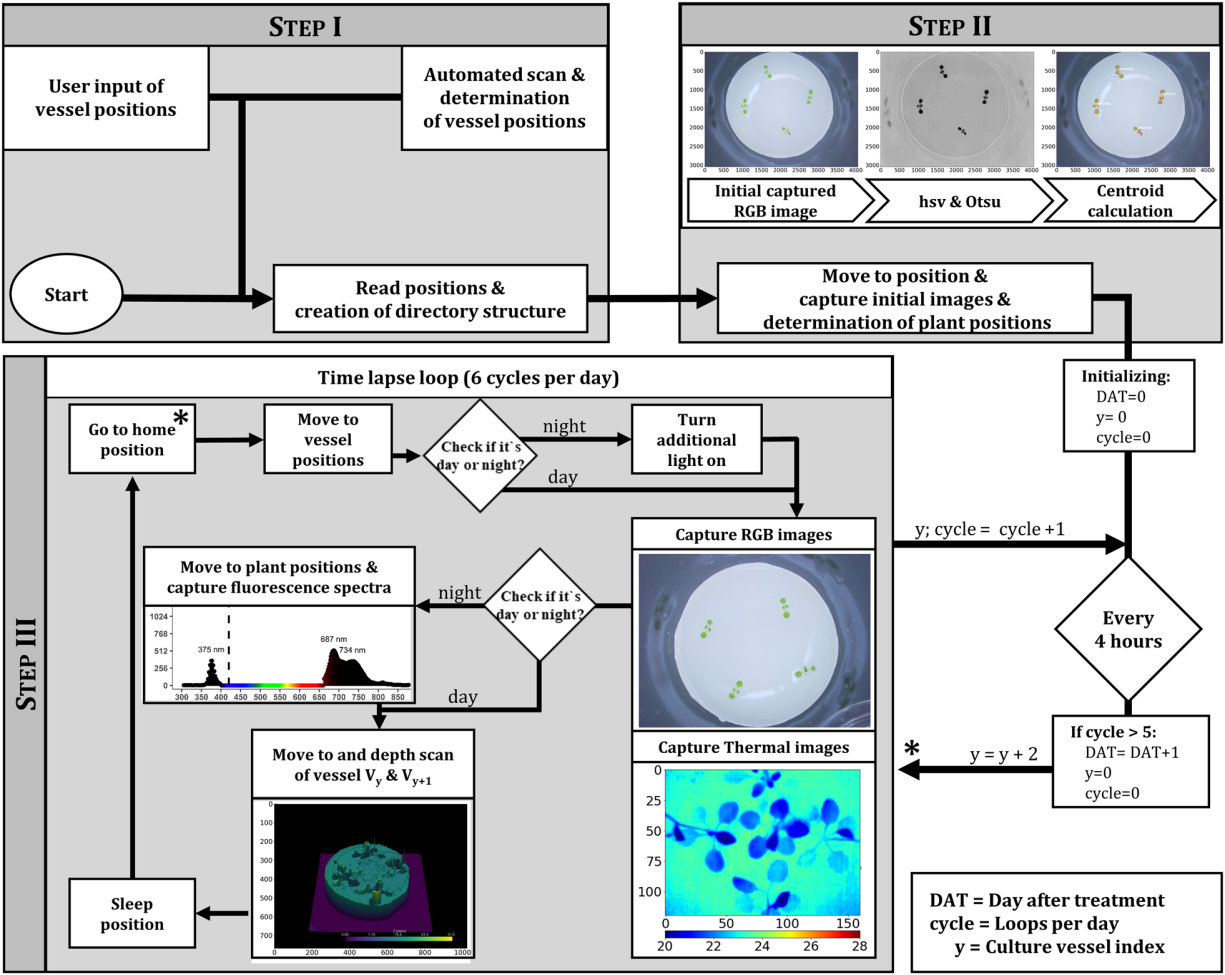
Flow chart of the three main steps of the automated phenotypic data acquisition (indicated in gray). In step I, the position of the culture vessel is determined, while in step II the initial images and the calculated plant positions are acquired. To determine plant positions, the original image was transformed to hsv-colorspace and the h-channel was segmented with Otsu-Method [20]. Four largest objects were selected as plant positions. Step III includes the actual time-lapse loop (start indicated by asterisk), where data of the four sensors are recorded. Detailed description in “Methods” section

**Fig. 4:**
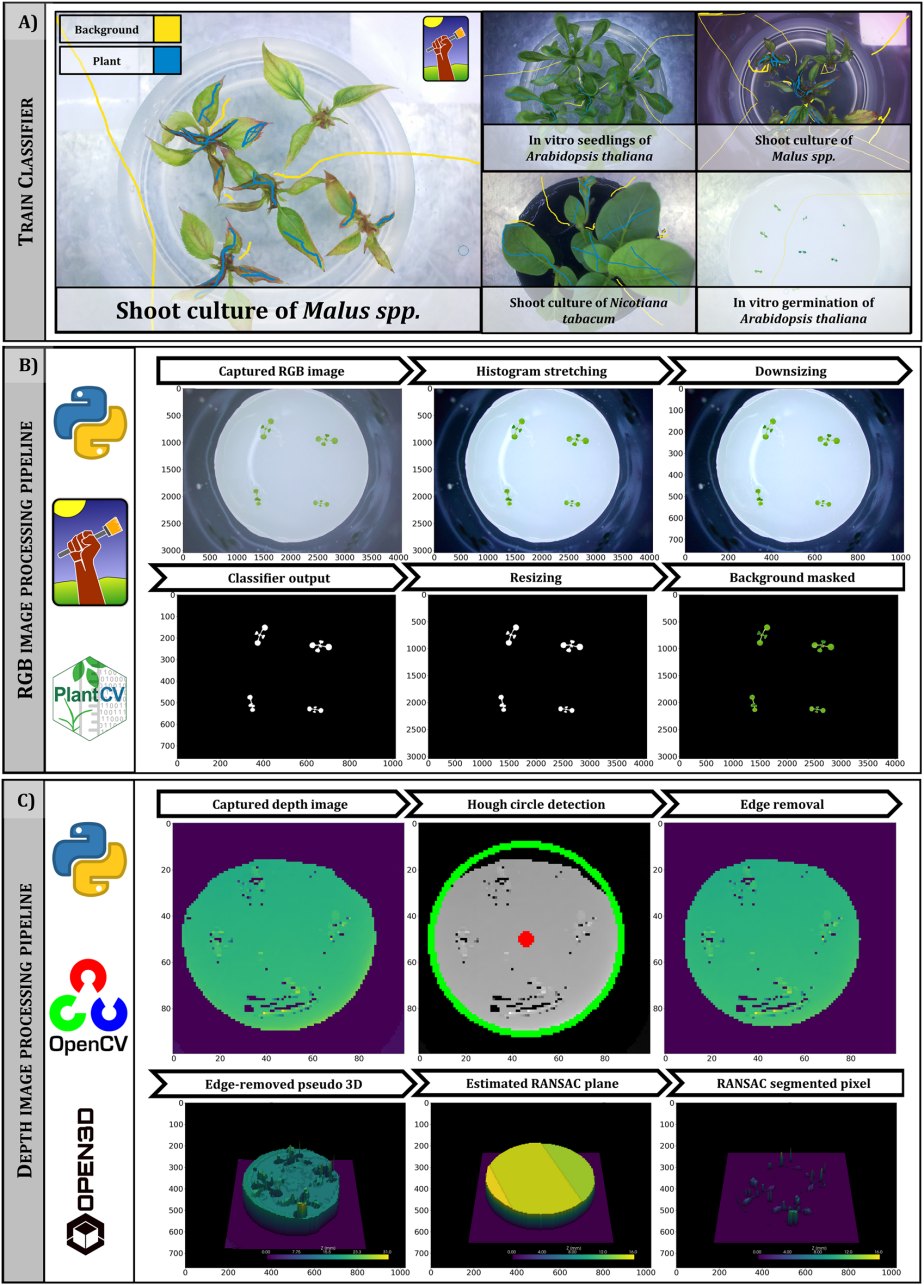
Overview of main data processing steps and used software packages to process the different types of acquired data. **A** A trainable Ilastik [21] classification model was trained to robustly cover the diversity of background (yellow labels) due to changing background and media color and diversity of foreground (blue labels) such as different plant species appearance and explant color changes during cultivation. **B** RGB image processing pipeline was developed in Python [22] with OpenCv [23] and PlantCv [24] for batch processing and including the ilastik classification model headless for segmentation. Upper row: RGB image processing workflow included an automated brightness and contrast adjustment by histogram stretching, down-scaling of image resolution from 4054 px × 3040 px to 1014 px × 760 px. Lower row: The trained classifier predicted binary mask of plant pixels rescaled to the original image resolution and applied to the original image for background removal. Exemplary images from monitoring of *A. thaliana* (Trial A). **C** For depth data processing, Python with Open3D [25] was used as an essential component to perform RANSAC [18]-based segmentation. Depth data of in vitro grown *A. thaliana* seedlings (Trial A). Upper row: Day 0 (Media with 10 day old, small seedlings), Hough Transform circle detection [26] and edge-removed depth image. Lower row: Pseudo 3D visualization of depth data of Day 11, estimated RANSAC plane and plant height surface corrected by estimated RANSAC plane at Day 11. Detailed description in “Methods” section

The phenotyping system was design as a scanning imaging system (xyz-gantry) for an autonomously operating acquisition of multi-sensor data, including RGB, thermal, depth and spectral data with specifically developed illumination (Fig. 2). Essential steps of continuous data acquisition with non-imaging and imaging sensor technologies were developed (Fig. 3). We could experimentally determine the technical repeatability for xy-axis with a MAE_X_ of 0.23 mm and a MAE_Y_ of 0.08 mm of the repositioning over 16 days via RGB image analysis of a reference object (described in detail “Methods” section). For the z-axis, a technical repeatability with a MAE_Z_ of 0.09 mm was obtained by using the calibrated laser distance sensor. For data segmentation a RGB image processing pipeline based on a random forest classifier and a depth image processing pipeline based on RANSAC [18] were newly established (Fig. 4).

### Optical properties of culture vessels

**Fig. 5:**
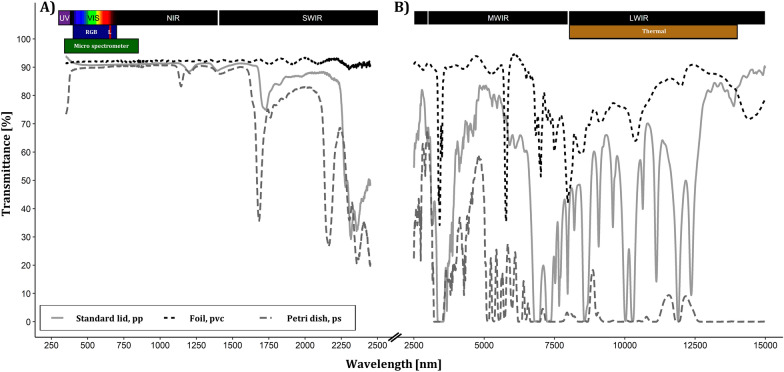
Spectral transmittance of culture vessel closures. Transmittance was measured with **A** an UV/VIS/NIR spectrometer and **B** with a FT-IR Spectrometer. Three independent replicates were measured and mean spectra per lid material are shown. Colored rectangles indicate waveband regions and spectral sensitivity of the sensors (according to the manufacturer’s specifications) installed in the phenotyping platform (blue, RGB camera with Sony IMX 477 sensitivity: 400 to 700 nm; green, micro spectrometer sensitivity: 340 to 850 nm; red, L, Laser distance sensor emission wavelength: 655 nm; brown, thermal camera sensitivity: 8000 to 14,000 nm)

In order to ensure high quality data acquisition for the four sensors and their respective spectral working ranges, spectral transmittance measurements were conducted from the ultraviolet (UV) to the long wavelength infrared (LWIR) region of three possible culture vessels and lids (Fig. 5). The polypropylene lid and the polystyrene Petri dish represented the standard culture vessels, while the polyvinyl chloride foil was included as an alternative sealing.

While all of the three tested sealings had high transmittance (> 91%) in the visible spectrum (VIS) (Fig. 5A), the tested materials differed strongly in the proportion of transmitted diffuse light (Table 1). The ratio of both is described by the Haze index, according to standard test method ASTM D1003 [19], thus representing an indicator for light scattering effects and visual perception by camera chips. Haze index should be kept to a minimum in imaging situations to maintain sharpness and clarity of the monitored object. The high Haze index of 34.2% excluded the standard polypropylene lid for being used in the phenotyping approach, while the polystyrene Petri dish and the PVC foil provided a clear VIS transmittance indicated by much lower Haze indices of 0.5% and 1.4%, respectively. In addition, a low to medium mean transmittance in the thermal range of 1.9% for the Petri dish and 50.6% for the polypropylene lid was determined (Fig. 5B). However, the foil still perceived a mean transmittance of 78.4% in thermal region. Thus the PVC foil was most suitable as a sealing system for imaging approaches for plant tissue culture, neglecting other not tested physical properties.

**Table 1:** Optical characteristics of in vitro culture vessel sealings

Culture vessel sealing	Replicates	Total transmittance [%]		Diffuse transmittance [%]		Haze index [%]		Thick-ness [µm]
		Average	SD	Average	SD	Average	SD	
Standard lid, PP	3	91.3	0.3	3.3	0.9	34.2	0.9	200
Foil, PVC	3	92.6	0.3	1.4	0.2	1.4	0.2	20
Petri dish, PS	1	91.2		0.6		0.5		900

*PP* polypropylene, *PVC* polyvinyl chloride, *PS* polystyrene

Measured with UV/VIS/NIR Spectrometer (PerkinElmer Lambda 1050) in 5 nm intervals from 380 to 780 nm according to standard test method ASTM D1003[19]

Haze index [%] = ((Diffuse transmittance/total transmittance) − rel. scattered transmittance by the system) × 100

### Collection of representative phenotypic data of plant in vitro cultures

The following results derived from automated data acquisition by the phenotyping system “Phenomenon” according to Fig. 3, which included an automated sequential approach of culture vessel positions and acquisition of multi-sensory data over weeks. Exemplary data analysis were conducted by automated data processing pipelines presented in Fig. 4, where automated segmentation of RGB and depth data were performed.

#### RGB data—Exemplary data analysis and validation of RGB image processing pipeline

**Fig. 6:**
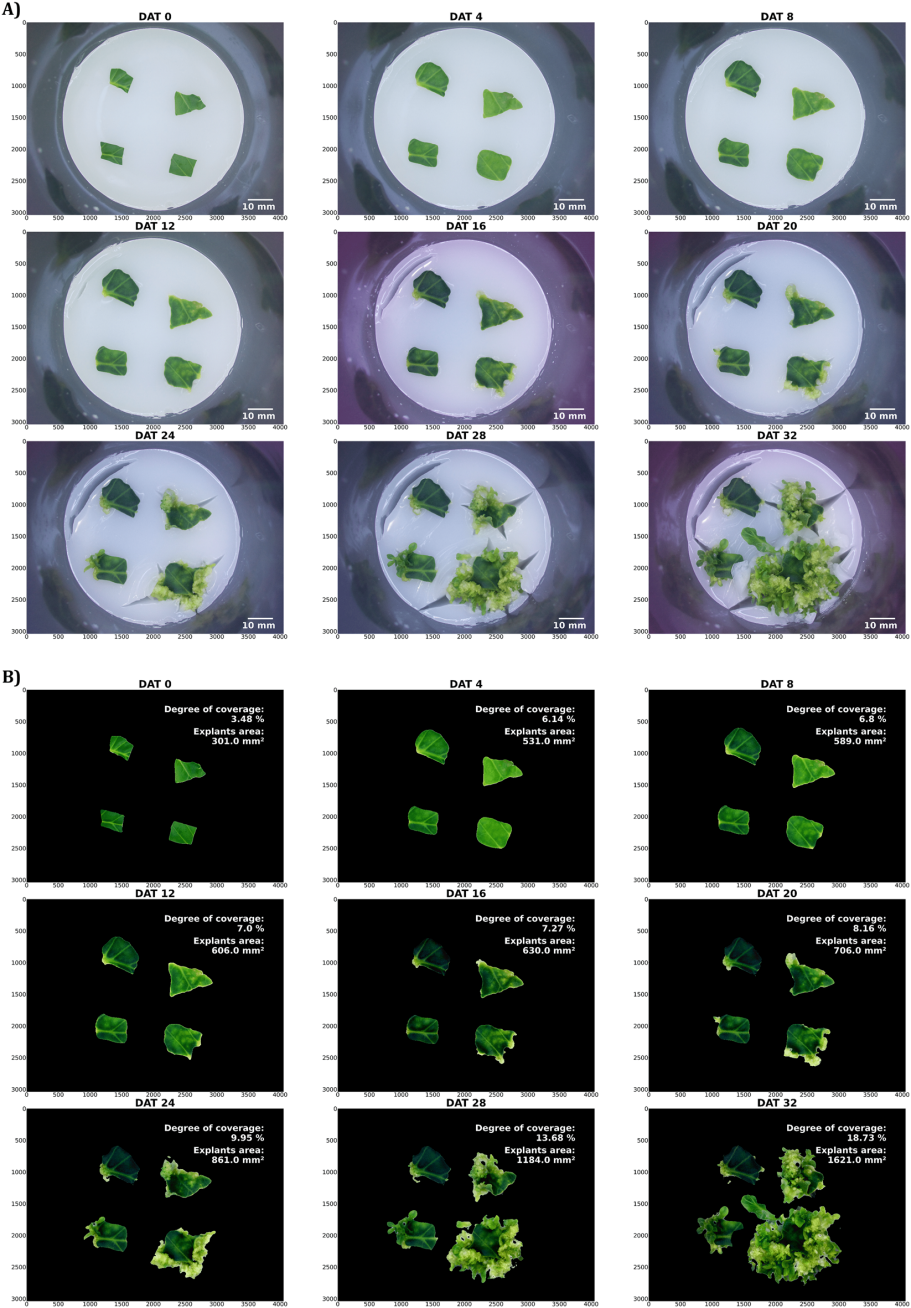
Monitoring of shoot regeneration of *N. tabacum* leaf explants. **A** RGB raw images and **B** processed images with the RGB imaging processing pipeline. *N. tabacum* leaf explants were placed on regeneration medium and developing adventitious shoot clusters were recorded over 32 days after treatment (DAT). Degree of coverage was calculated as the sum of plant pixels divided by total number of pixels within an image. For determination of explants area, sum of plant pixels was multiplied by pixel-metric-conversion factor. Time lapse video of *N. tabacum* regeneration is provided in Additional file 1

Several in vitro phenotyping approaches were conducted with the “Phenomenon” system to demonstrate its full potential, including different plant species (*Arabidopsis thaliana*, *Nicotiana tabacum* and *Malus domestica*—data not shown) and developmental phases (in vitro germination, shoot and root regeneration and shoot multiplication). Figure 6 demonstrates the regeneration of adventitious shoots of *N. tabacum* from leaf explants monitored (6 images per day) over 32 days after treatment (DAT) and the output of the automated RGB processing pipeline of Fig. 4. This experiment clearly illustrated the segmentation challenges for image analysis such as similar color appearance of developing cell and organ types such as callus or roots and the cultivation medium, medium adhering to the plant cluster and camera specific changes in color balance. Additional file 1 contains a complete time-lapse video of one of the culture vessels. Regardless of the challenges mentioned, this video demonstrates the great potential of “Phenomenon” in terms of time series observations.

**Fig. 7:**
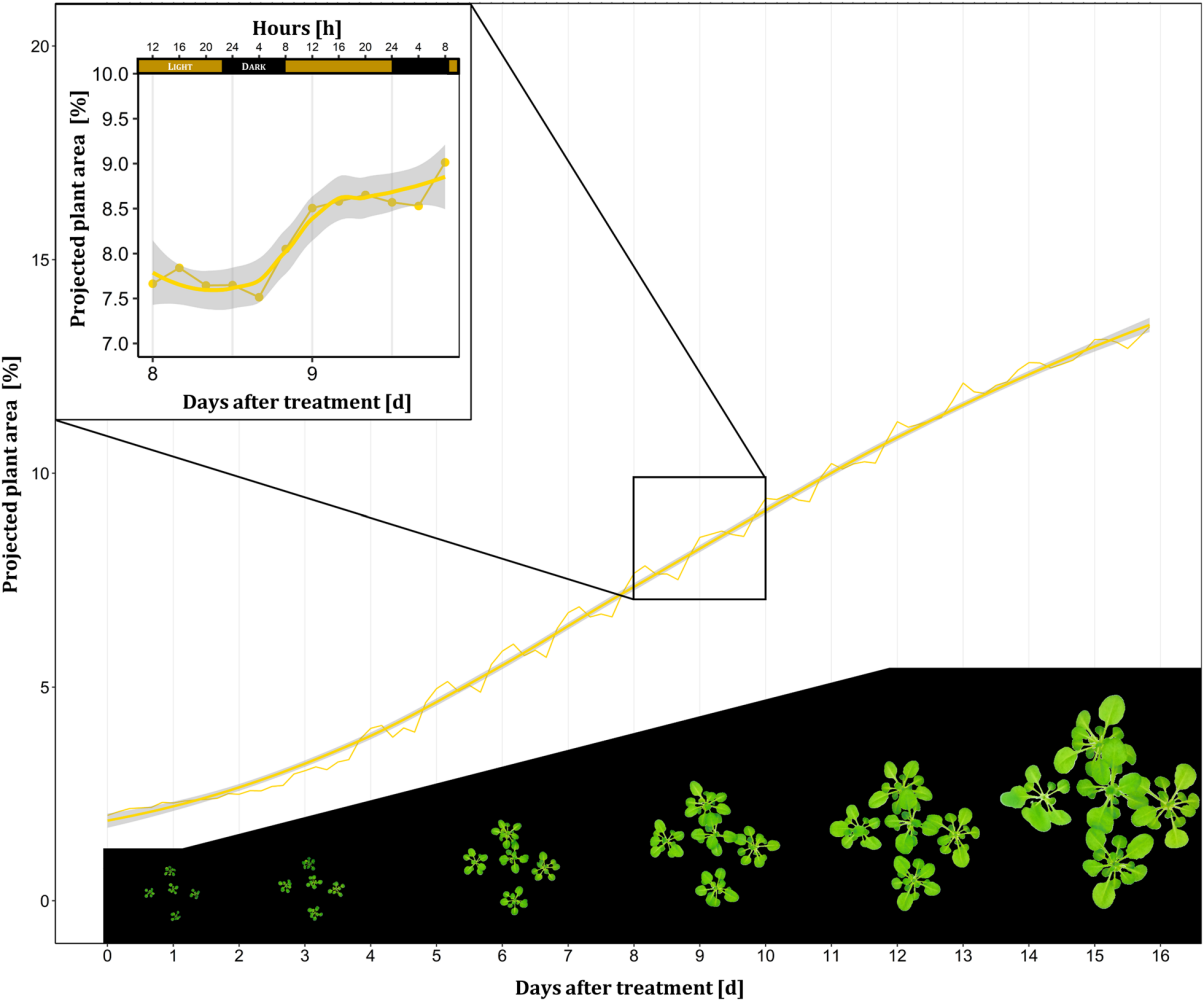
Exemplary growth curves of one culture vessel containing five *A. thaliana* seedlings (Trial B) expressed as projected plant area. Projected plant area was calculated as the sum of plant pixels divided by total number of pixels within an image. Yellow smoothed line plot, method = logistic regression, gray indicates confidence interval borders α = 0.95. Six images per day over 16 days resulted in a total number of 96 images per growth curve. Left corner highlights a closeup showing the diurnal rhythm of plant growth. The bottom part contains segmented images of 0, 3, 6, 9, 12, 15 DAT (days after treatment). Time-lapse video of *A. thaliana* (Trial B) is provided in Additional files 2, 3

As second demonstration of the functions of our phenotyping system, Fig. 7 shows the whole life-cycle monitoring of *A. thaliana* in vitro (seedling to flowering plant) and the calculation of growth performance metrics. Time-lapse videos of *A. thaliana* monitored over 16 days are provided in Additional files 2, 3.

**Fig. 8:**
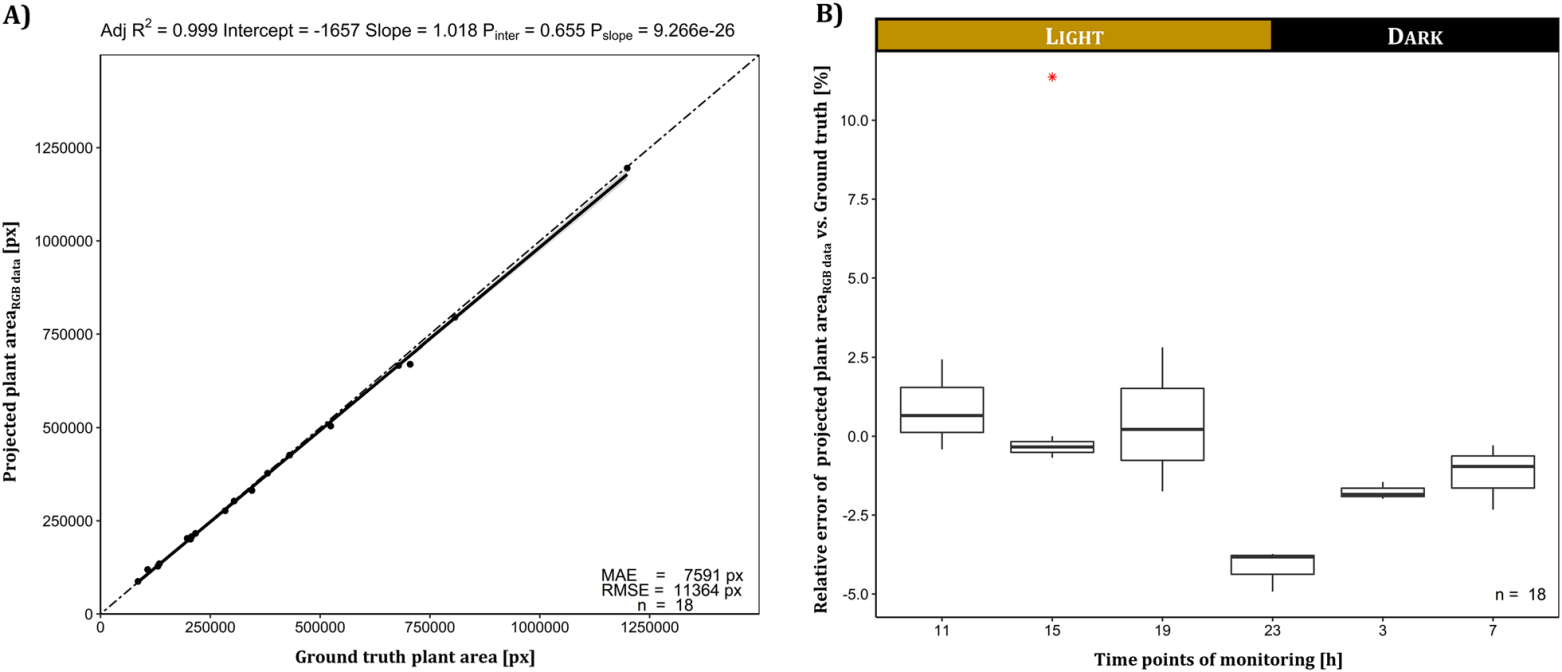
Characterization of the segmentation performance of the RGB image processing pipeline from 18 randomly selected images of the *A. thaliana* Trial A dataset. **A** Linear regression of projected plant area vs. ground truth plant area. The regression line is colored black, while the angle bisector line is drawn two-dashed. Gray indicates confidence interval limits at α = 0.95. Adj R^2^ denotes the coefficient of determination adjusted according to Yin and Fan [27], while P_slope_ and P_inter_ represent p-values of the coefficients for the intercept and slope determined by simple T-test. MAE and RMSE indicate the mean absolute error and the root mean square error of the projected plant area. **B** Relative error of plant area projection for different acquisition time points (23, 3 and 7 o’clock represented night conditions) calculated from 18 randomly selected images of the *A. thaliana* Trial A dataset. First manual annotated image was identified as an outlier marked as red asterisk

The validation of the projected plant area obtained as one output from the RGB image processing pipeline by relating it to the projected plant area determined by manual annotation of plant pixels (ground truth) indicated a high R^2^ of > 0.99 (Fig. 8A). The automated classification approach overestimated the plant area with an average error of 7591 px. The relative error of the different acquisition time points (Fig. 8B) indicated a slightly higher overestimation at day time images, while an underestimation occurred for night time images (with highest error at 23 o’clock), resulting in a mean relative error (MRE) of 0.37% overestimation. To quantify the classification performance, confusions statistics of 221,834,880 pixel pairs were conducted and disclosed a classification accuracy of 97.7%, a sensitivity of 97.7%, a specificity of 96.9% and a precision of 99.9% for the segmentation of the proposed RGB image processing pipeline.

#### Depth data—Exemplary data analysis and validation of depth image processing pipeline

**Fig. 9:**
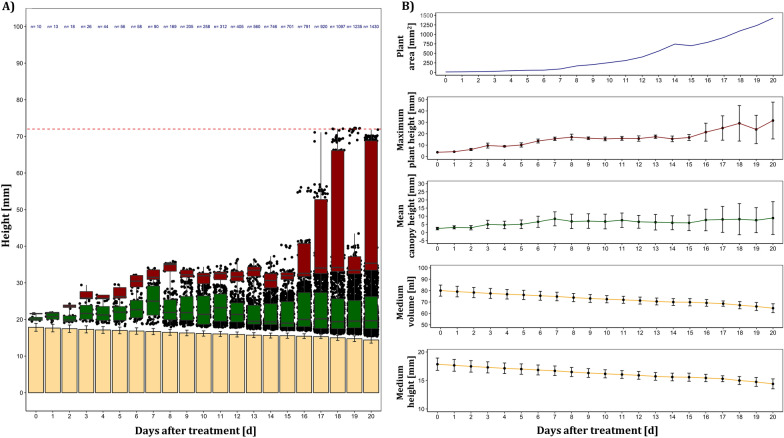
Exemplary depth data analysis of a culture vessel with four *A. thaliana* seedlings grown in vitro for 21 days (Trial A). **A** Yellow bar plot displays calculated media height (Mean ± SD), while black dots indicate sensor values of plant pixels after segmentation and colored boxplots indicate values for the calculation of the two digital parameters mean canopy height (green) and maximum plant height as mean of the upper 10 percentile (red). Red dashed line represents the maximum height of the sensor reliable distance (< = 72 mm) and the amount of plant pixels after segmentation was colorized in blue. **B** Individual plots of the dynamic behavior of the calculated digital parameters with equal color code and depicted as means ± SD. The calculation of medium volume and all other parameters is described in “Methods” section. 10,000 data points for each date were processed from depth scans of an area of 100 mm × 100 mm with a scan pattern of 1 mm × 1 mm. Depending on the necessary segmentation for the calculation of the individual parameters, a corresponding proportion of the 10,000 data points was included in the analysis

The first report of depth data acquisition and analysis in plant in vitro culture is illustrated in Fig. 9A using *A. thaliana* as an example. It included the calculation of important biological parameters from the depth data set to monitor culture medium height, culture medium volume, mean canopy height, maximum plant height and degree of coverage (Fig. 9B). Figure 9 clearly demonstrates a height and volume reduction of culture media, while in plant growth related parameters a height increase was notable. Corresponding RGB images revealed first signs of flower induction of the *A. thaliana* seedlings at DAT 12, and an associated increase in maximum plant height was to be seen in depth data at DAT 16. In addition, also the variance in average canopy height increased at DAT 16.

**Fig. 10:**
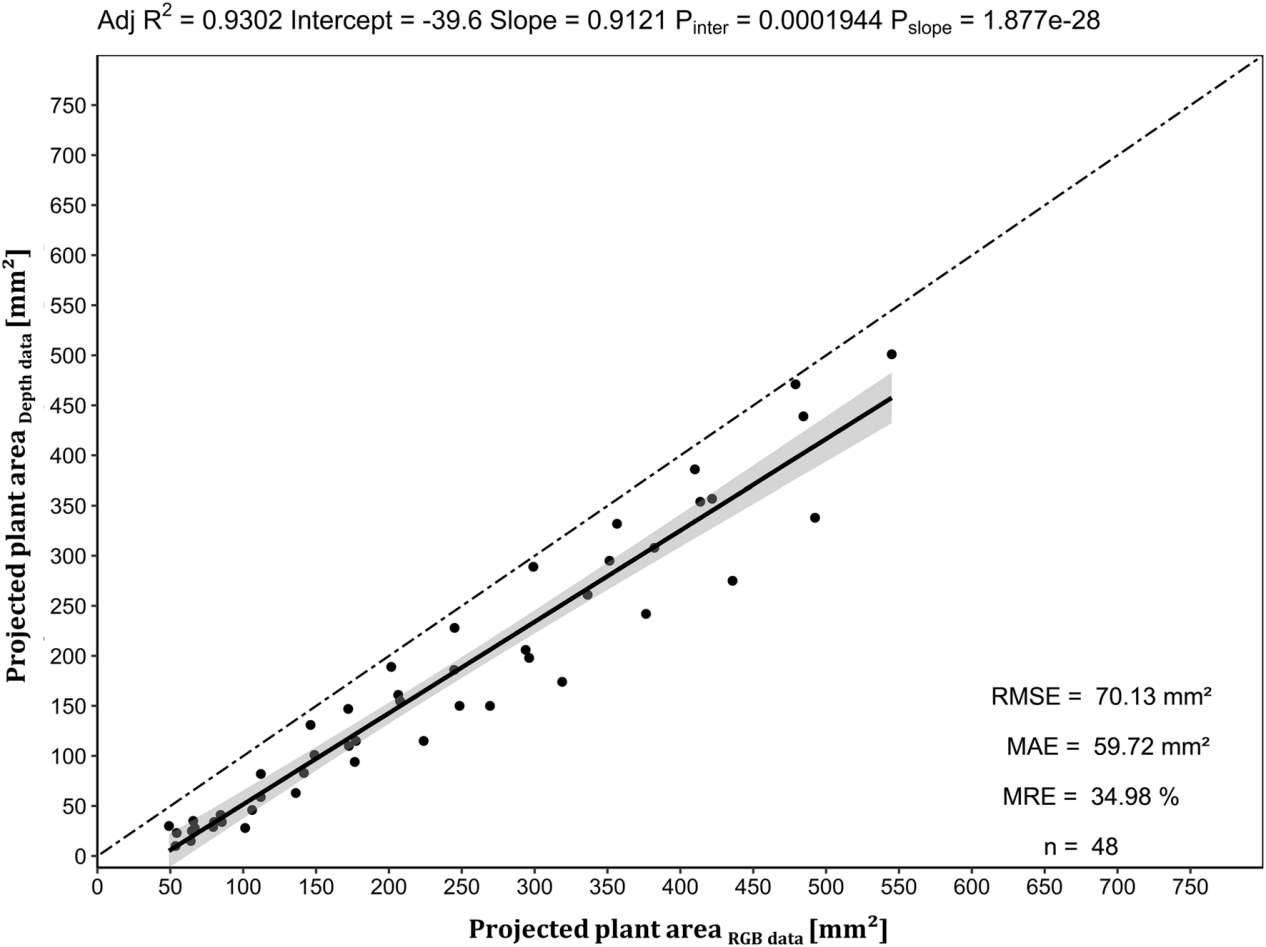
Linear regression of projected plant area as output of the depth data pipeline vs. projected plant area obtained from the RGB data pipeline (ground truth). The regression line is colored black, while the angle bisector line is drawn two-dashed. Gray indicates confidence interval limits at α = 0.95. Adj R^2^ denotes the coefficient of determination adjusted according to Yin and Fan [27], while P_slope_ and P_inter_ represent p-values of the coefficients for the intercept and slope determined by simple T-test. MAE, MRE and RMSE indicate the mean absolute error, mean relative error and the root mean square error of projected plant area. Sampling (n) was formed out of 12 images from four different culture containers and 12 time points respectively (DAT 0–DAT 11)

Comparing the projected plant area obtained from the RGB processing pipeline (assumed as the ground truth) with the projected plant area as output from the depth processing pipeline, a high correlation, expressed in an R^2^ of 0.93 was observed with an average underestimation of 59.7 mm^2^ (MAE) of plant area determined by depth data (Fig. 10). The mean relative error (MRE) revealed that the depth data processing pipeline projected the plant area by 65% compared to RGB processing pipeline, meaning 35% of plant pixels were systematically not detected by the sensor or have been removed due to segmentation. This can be considered as a rough estimator of how accurately the different sensor technologies (RGB camera vs. scanning laser distance sensor) detect plant pixels, including the errors derived from segmentation and differences in object area representation by the two sensor technologies.

#### Spectral data—Exemplary data analysis and validation of detection spot size

**Fig. 11:**
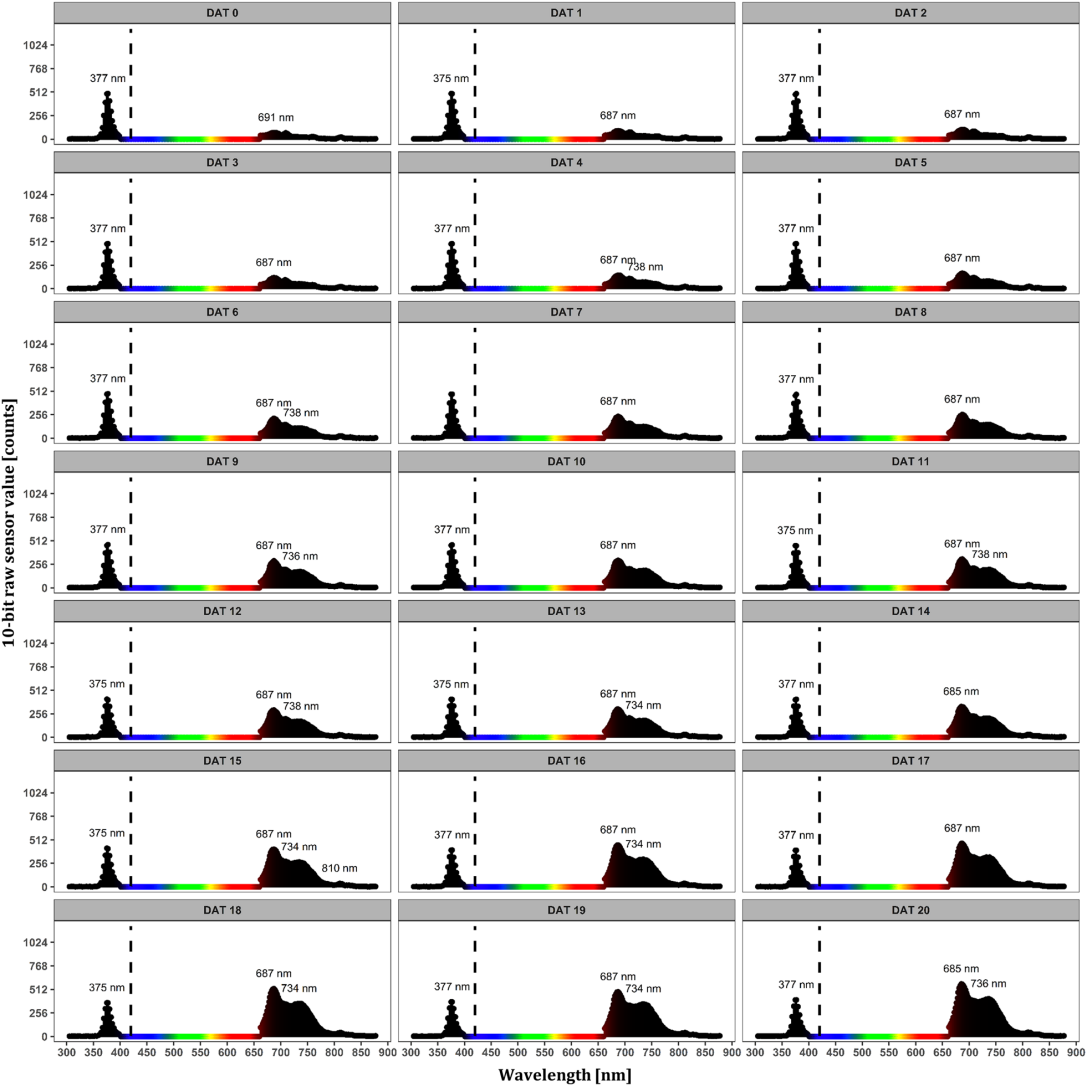
Exemplary determination of dynamic chlorophyll fluorescence monitoring of one of four *A. thaliana* seedlings grown in vitro for 21 days (Trial A). The first peak at 375 nm can be assigned to the excitation light provided by UV LEDs imperfectly blocked by the long pass filter at 420 nm (black long dashed line). The region from 400 to 660 nm has been masked for simplified representation. Emission peaks in the region from 660 to 780 nm indicated the two typical maxima of the chlorophyll fluorescence, derived from PSII (F690) and PSII and PSI (F730). Micro spectrometer integration time was set to 300 ms

An automated and dynamic monitoring of the chlorophyll fluorescence signature of an *A. thaliana* seedling cultured in vitro over 21 days is illustrated in Fig. 11. Excitation light emission maximum at 375 nm as well as a sequential increase in the fluorescence signal depending on the plant growth were evident. Typical emission maxima derived from the reaction centers of the photosystem (PS): mainly PSII (F690) and PSII and PSI (F730) were detected. Furthermore, we have determined the diameter of the detection spot of the modified spectrometer to be 23 mm (detailed description in “Methods” section).

#### Thermal data—Exemplary data analysis and validation

**Fig. 12:**
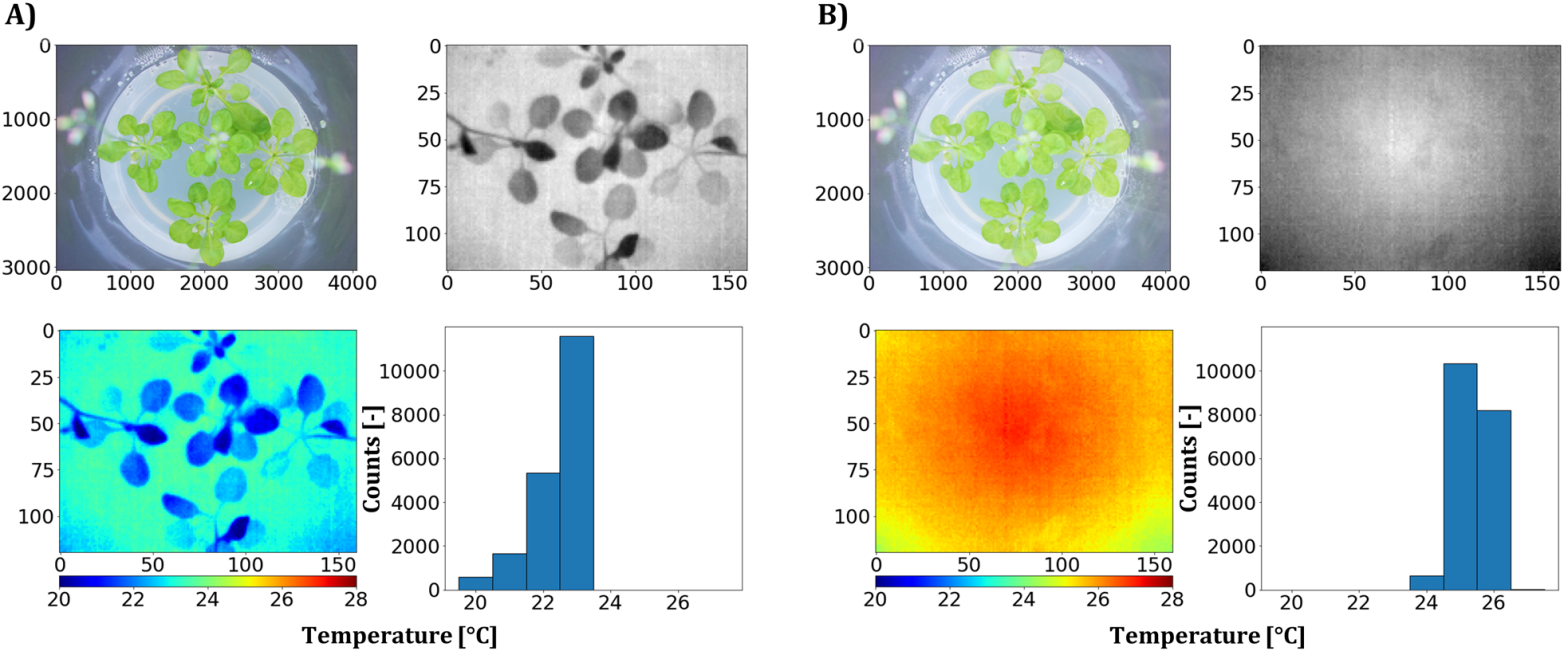
Exemplary thermal imaging of in vitro cultivated *A. thaliana* seedlings (Trial A). **A** Left side demonstrates imaging without the foil that was used to seal the culture vessels, while **B** the right side shows acquired sensor data through the foil. The respective RGB images are shown and thermal data are presented as grayscale and false color images and corresponding histograms

Thermal imaging of in vitro cultivated *A. thaliana* seedlings was attempted, but faced the challenges of the special imaging situation (Fig. 12). When captured without the sealing foil, the thermal images (Fig. 12A) of culture vessels at a room temperature of 25 °C (day) and placed on a bottom-cooled shelf surface with a temperature of 21.5 °C, revealed reasonable absolute values of thermal data, as indicated in the corresponding histogram. However, thermal images taken through the sealing foil (Fig. 12B) only allowed a weak separation between plant and background pixels and the corresponding histogram indicated an increase in radiometric data.

## Discussion

To the best of our knowledge, this is the first report of a multi sensor phenotyping system, based on an xyz-gantry that is capable of autonomous acquisition of relevant sensor data of plant in vitro cultures. The selection of exclusively low-cost hardware (Table 2) and open-source software components accessible via the GitHub repository [28] enables other researchers to rebuild the “Phenomenon” system and to benefit from it in science, education and commercial micropropagation.

**Table 2:** Main system components and costs

Description	Quantity	Hardware	Price
G420 Long pass filter	1	Dielectric coated long pass filter	40 €
PCB manufacturing cost	1	Ring light PCB and a Circuit PCB	40 €
Various LEDs	48	Standard 5 mm LEDs (375 nm, 6500 K, 700 nm)	40 €
RGB camera	1	Raspberry Pi Camera High quality	50 €
3D filament, cable chain, limit switch	1	Small mechatronic parts	100 €
Network communication	1	Router & PoE-Switch	100 €
Single-board computer	2	Raspberry Pi 4B & PoE-Shield	120 €
Z-axis with Nema 23 Stepper motor	1	OpenBuilds Linear Actuator	160 €
Thermal camera	1	PureThermal 2 & Lepton 3.5	250 €
Micro spectrometer	1	Mini-Spectrometer C12880MA	350 €
Low distortion lens	1	Edmund Optics 6 mm lens	400 €
Xy-gantry with 24 V power supply and 3×Nema 17 Stepper motor	1	OpenBuilds ACRO 1515 60" × 60"	410 €
Laser distance sensor	1	OD-Mini OB1-B100	1000 €
Total			3060 €

As proposed by Dhondt et al. [29] phenotyping systems can be defined by system properties like throughput, resolution and dimensionality. With the current setup, we reached a throughput of multi-dimensional data (RGB, depth, spectral, thermal) at a macroscopic resolution for ten culture vessels per day. Therein, the main limiting factor was the time-consuming process of depth data scans (45 min per vessel; compared to RGB and thermal image and spectral point measurements with only a few seconds per vessel) and system dimensions restricting the working area. Low cost imaging depth sensors based on “time-of-flight” principle (ToF) such as Pieye Nimbus 3D or Onion tau could reduce substantially the acquisition time of depth images. However respective sensors need to tested how they perform under the highly challenging imaging conditions (Fig. 1) of plant in vitro cultures. Nevertheless, a large-scale application can be achieved with minimal effort and costs if the robot system working area is scaled up to a whole shelf.

We aimed at monitoring plant in vitro cultures with minimal invasiveness, consequently phenotyping took place dynamically within in the cultivation of in vitro cultures, instead of monitoring open culture containers under laminar flow to ensure aseptic conditions. Non-destructive phenotyping approaches where optical sensing happens trough the vessel encounter a challenging imaging situation (Fig. 1) and could be solved in parts by the technical design of “Phenomenon”. However, three modifications were necessary to increase sensor data quality: (i) The culture vessels were placed on a bottom-cooled surface to avoid condense water formation. Bottom cooling systems are widely applied in tissue culture to reduce the relative humidity in the vessels and thereby increasing plant quality, but in case of rose roots also slowed down the growth of cultures due to the lower temperature [30]. (ii) The plastic lid was substituted by a PVC foil to maintain a clear and undistorted view, evidenced by the Haze index (Table 1) and to increase the spectral transmittance in the thermal region (Fig. 5). This also affected the gaseous exchange capacity of the culture containers, which was notable by increased evaporation of water from the culture media. The use of the foil also prevented condense water formation, when the bottom was not cooled. Thus, this intervention might be sufficient. Nevertheless, future research should address an optimization of the culture vessels/lids to enable proper imaging sensor application in vitro. (iii) The supplementation of the culture media with TiO_2_ allowed a detection of the surface of the normally semitransparent media with the laser distance sensor (detailed description in “Methods” section). TiO_2_ had already been used in plant in vitro culture due to an antimicrobial activity induced by UV excitation [31]. However, beneficial or cytotoxic effects of TiO_2_ nanoparticles (NPs) in particular, are currently under research and most likely will be depending on the dose and UV exposure time [32]. TiO_2_ NPs had no negative effect on the growth of soybean seedlings in vitro at concentrations of 10 and 100 mg L^−1^ TiO_2_ NPs, but slightly reduced fresh mass and root growth at 1000 mg L^−1^ TiO_2_ NPs [33], suggesting a reduction of the TiO_2_ concentration in the culture media for upcoming experiments.

The **automated scanning imaging system “Phenomenon”** based on a belt-driven xy-gantry and screw-driven z-axis was specified by the manufacturer to provide an accuracy of 0.1 to 0.2 mm for the xy-axes and 0.05 to 0.1 mm for the z-axis. Experimentally, we determined the technical repeatability for MAE_X_ of 0.23 mm, MAE_Y_ of 0.08 mm and MAE_Z_ of 0.09 mm. Considering the fact that an exclusively low-cost phenotyping system was intended, a sufficient technical repeatability was achieved for consistent data acquisition.

RGB data acquisition was conducted with a low-cost **RGB sensor** equipped with a low distortion lens to minimize the error of projection. This error resulted in a distortion of the projected plant area at the edges of the image compared to the midpoint. Furthermore, the estimation of plant area, i.e. of an 3D object, with a 2D sensor without a telecentric lens can be put into question. However, plant cultivation in multi-shelf systems (Fig. 2) with a distance between the cultivation area and the illumination of 400 mm, limited not only the application of optics greater in size but also the selection of other sensor technology by their optical specifications (minimum working distance; MWD < 150 mm).

In this study, we demonstrated a successful implementation of a scanning laser distance sensor resulting in a **depth image** of plant in vitro cultures for the first time. Novel relevant traits of micropropagated cultures like medium height and deduced from this medium volume, average canopy height and maximum plant height could be quantified and will be validated in upcoming experiments. We showed a reliable application of this technology (Figs. 9, 10, Additional files 4, 5 and 6), but the reflection-based time-of-flight sensor failed, if the reflection surface was tilt with a higher angle (upwards growing leaves) and at the upper part of depth images, where the emission beam was inside and the detector side of the sensor still outside of the culture vessel (Fig. 4: missing part of detected Hough circle). In addition, it is worth mentioning that the detection error of the reflection-based sensor could be due to the fact that the emission wavelength of the laser distance sensor hits the absorption of the plant pigments at 655 nm. Therefore, depth sensors with spectral detection range in near infrared might be superior due to the higher reflectance signal derived from the red edge shape of the plant spectra.

The second novelty was the proof-of-concept for applying a low-cost **micro spectrometer** to determine spectral signatures, offering great potential for monitoring the stress status of in vitro cultivated plantlets. The point measuring device was limited in spatial resolution due to the detection spot size of around 23 mm (Additional file 7). Reflection-based measurements were therefore not exclusively-plant-specific. However, the fluorescence signature reflected plant specific peaks (Fig. 11). Known stress indices, like F690/F740 as a chlorophyll content estimator [34, 35]—can be calculated from the fluorescence spectra on an explant base and their potential use to detect early stress responses opens new ways in in vitro stress screenings, for example.

Leaf temperature quantification of micropropagated plants by **thermal imaging** approach was already investigated by Ibaraki and Gupta [9], but so far only after their transfer to ex vitro conditions. Thermal imaging of plants is widely used to estimate evapotranspiration-based parameters like water loss, water stress indices or stomatal conductance [9, 36, 37]. We could disprove the assumption that thermal imaging of in vitro cultures is impossible, even if data quality was limited in terms of contrast (Fig. 12). By using the PVC foil, we improved the average transmittance in the thermal waveband up to 78.4% (Fig. 5), but still absorption and reflection occurred and reduced the quality of the sensor data (Fig. 12). The increased mean temperature of explants imaged through the foil might be due to sensor self-reflection compared to the imaging without foil. Whether temperature differences between plants due to evapotranspiration can be quantified by thermal imaging of high humidity culture vessels (93 to 97% RH with bottom cooling [38]) remains to be answered.

The validation of the **RGB image processing pipeline** demonstrated the power of digital image analysis accomplished through successful segmentation. Figure 7 could demonstrate the potential for researchers to compare treatments, such as different media compositions, or to track small leaf movement like the diurnal growth rhythm. A robust and specific segmentation covering the required range of the imaging situation was only possible by a trainable segmentation model. Despite the acceptance of evoking errors by the use of reduced resolution images as an input of the segmentation model, a nearly perfect segmentation was achieved as indicated by the high coefficient of determination of R^2^ of > 0.99 referred to manual annotation of plant pixels. Confusions statistics revealed an even higher accuracy of 97.7% of classification compared to the study of Mestre et al. [8] reaching an accuracy of 96.9% although they used multidimensional data (RGB, NIR) as input for a random forest classifier to segment in vitro grown *Nandina domestica* explants. Main classification errors originated from overestimation of leaf borders by the automated RGB image processing pipeline compared to the ground truth segmentation and from false-positive classification due to root greening. The time point-dependent performance of the segmentation during the day (Fig. 8B) can likely be attributed to insufficient illumination of tiny plant structures such as leaf petioles, where the average light intensity captured by the camera revealed minimum residual light at the time point of the greatest underestimation (23 o’clock). Interestingly, a correlation coefficient of 0.75 indicated a strong correlation between the absolute classification error and the average mean intensity of the RGB images, explaining the difference at night time points where variations in residual light intensity occurred due to the switch timing of the tube fluorescent lamps (data not shown). Projected plant area can be used as good estimator for biomass as a key performance parameter of plant in vitro cultures as shown by Faragó et al. [6] who identified a coefficient of determination of R^2^ = 0.99 between *A. thaliana* digital rosette size and fresh mass. As a common issue of image analysis, it might be questionable whether the probabilistic based random forest model or the human labeled classification better reflect the real ground truth of in particular imperfect-focused leaf borders. It has to be stressed, that for other growth habits, such as upright growth with several layers of overlapping leaves, it will be more difficult to correlate projected plant area and biomass, but in these cases additional information from depth data may be used to define additional covariates.

The **depth image processing pipeline** showed that segmentation of plant in vitro depth data over time requires a dynamic approach to accommodate changing processes like plant growth or culture media shrinkage via evaporation. The separation of background, culture medium and plant pixels was the main objective of the segmentation for calculating relative plant height, culture medium height, and accounting for tilts of the cultivation surface or the medium surface. An image registration approach of RGB (where a good segmentation was already achieved) and depth images was not satisfying due to the too different representation of objects by the two sensor technologies. A Random sample consensus (RANSAC [18]) algorithm fulfilled the requirements of the task and was able to dynamically and robustly detect the culture medium surface planes within the one-dimensional and therefore difficult to segment data. RANSAC is a robust method for an iterative determination of outliers from a mathematical model in an overdetermined data set. The RANSAC approach is commonly used in depth data segmentation of plants [39-41] and allowed the determination of relevant and novel digital features of plant in vitro cultures like culture medium height, mean canopy height, maximum plant height and plant area by depth data. However, limitations will arise when the culture medium surface is fully covered by plants and therefore, no longer represents the largest plane. The determination of the height of each explant inside the culture vessel is to be aimed, but requires connected compounds after segmentation. To estimate the quality of the representation of plants in depth images we selected the projected plant area as a basis of comparison between RGB and depth sensor data and corresponding pipelines, respectively. The high mean relative error (Fig. 10) demonstrated the limitations of the scanning laser distance sensor, as only two thirds of the projected plant area were represented in the depth data after segmentation. This error could be attributed to detection errors, unfavorable reflection due to inclined surfaces (e.g., leaves growing upwards) or water drops on plants, the low spatial resolution of the scan pattern (1 mm × 1 mm) or errors caused by segmentation. Nevertheless, depth data of plant in vitro cultures could be used to estimate plant biomass, especially when combined with projected plant area by RGB images. Furthermore, the determination of culture medium volume opens the possibility to collect new data of plant water uptake and evaporation from culture medium.

**Fig. 13:**
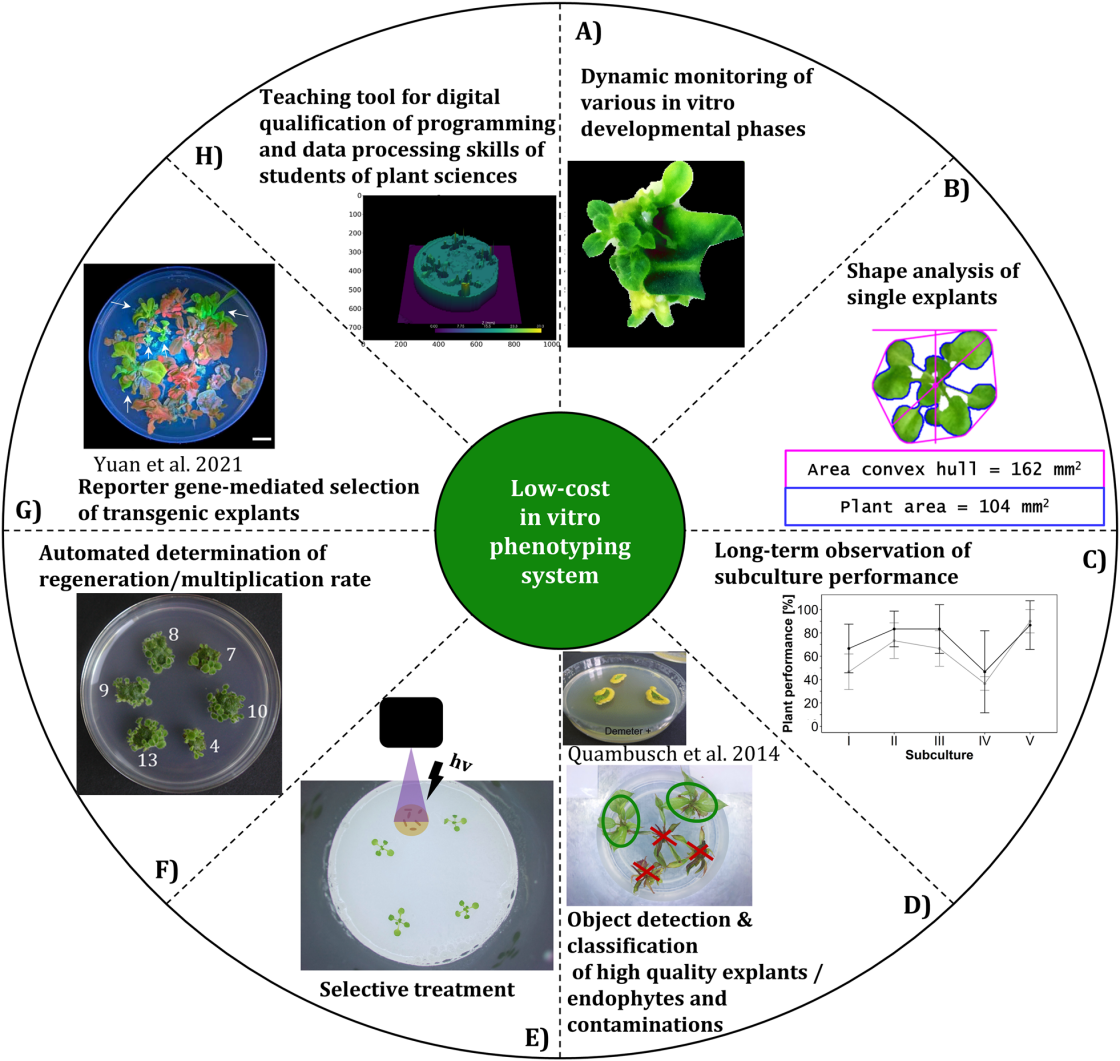
Potential applications of the automated low-cost phenotyping system in plant vitro culture. While the requirements for the use cases from **A**–**C**, **E** and **H** have already been met, further research is required for the use cases **D**, **F** to **G** (External images from Yuan et al. [42] and Quambusch et al. [44])

We have designed, constructed and tested a novel multi-sensor robot platform for phenotyping in plant in vitro cultures offering great potential for automatization of specific tasks in commercial micropropagation, but also offering new possibilities in research (Fig. 13A–C). The “Phenomenon” phenotyping system differentiates from existing in vitro monitoring approaches that focused primarily on shape analysis and the application of which was limited to *A. thaliana*, mainly. The system allows phenotyping of different species and different developmental phases in in vitro culture due to its customized and specific hardware design (Fig. 2). Repeated monitoring of individual cultures regarding their growth performance over several culture passages will reveal new insights into phenomena such as the habituation against phytohormones or seasonal variation of growth. Tracing back the development of individual explants over time pave the way for improvements in cultivation. Furthermore, the system could be used to optimize culture medium compositions like amount of plant growths regulators via an objective quantification of the plant phenotypic characteristics. A future perspective of sensor application in plant in vitro culture by automated imaging robots includes the construction of multi-sensor data sets to benefit from ever easier access to the power of artificial intelligence such as artificial neural networks as reviewed in Prasad and Gupta [12] for complex classification or regression tasks such as the detection of endophytes or the calculation of multiplication rates of plant in vitro cultures (Fig. 13D and F). Furthermore, robots may offer the ability to identify and treat explants which exhibit a low or high stress level after certain treatments or to early detect potential contaminations (Fig. 13E). A marker gene-free early selection of transgenic plant material as proposed by Yuan et al. [42] with the usage of new reporter genes such as eYGFPuv could be automated by the presented low-cost phenotyping system (Fig. 13G). Finally, we suggest using the system in teaching to promote digital skills of plant science students. Since it features low-cost, stand-alone and portable characteristics, it may provide students with handling and processing of multi-sensory phenotypic data (Fig. 13H) [43].

## Conclusions

We developed a novel low-cost multi-sensor automated phenotyping system for application in plant in vitro cultures. The unique hard- and software concept is characterized by using exclusively low-cost compounds and open-source-based software components. This allows remote and programming language-independent access to its functionalities, enabling plant scientists to benefit from the capabilities with minimal financial investment. Various sensor technologies were applied for the first time under these challenging culture conditions and were evaluated with respect to resulting data quality and feasibility with proposed data processing pipelines. We demonstrated the digital determination of relevant parameters such as projected plant area, average canopy height, and maximum plant height, which can be used as critical descriptors of plant growth performance in vitro. The initial exemplary demonstration of resulting data promises great potential. The technical realization of “Phenomenon” enabled phenotyping of plant in vitro cultures under highly challenging conditions and will lead to increased sensor application approaches for research and commercial propagation in upcoming years.

## Methods

### Adventitious shoot regeneration from *N. tabacum* leaf explants

From in vitro grown *Nicotiana tabacum* ‘Samsun’ shoot stock cultures, leaf explants (5 to 6 mm edge length) were prepared and four each were placed in four 500 mL polypropylene containers containing 80 mL MS medium [45] supplemented with 3% (w/v) sucrose, 0.75% Plant agar (w/v) (Duchefa, Harlem, The Netherlands), 4.44 µM 6-benzylaminopurine (BAP) and 1 g L^−1^ titanium dioxide. The pH of the medium was adjusted to 5.8 prior to autoclaving at 121 °C for 15 min.

### Seedling growth of *A. thaliana*

*Arabidopsis thaliana* Col-0 seeds stored since 2018 at 4 °C were surface-disinfected using 70% (v/v) isopropanol for 30 s, followed by 2% (v/v) sodium hypochlorite plus Tween 20 for 5 min and rinsing three times in water. The seeds were germinated for 10 days at 24 °C in Petri dishes containing plant growth regulator-free B5 medium [46] with 1.5% (w/v) sucrose and 0.8% (w/v) Plant agar at pH 5.8. Ten days old uniform seedling were transferred to the same medium but supplemented with 0.1% (w/v) titanium dioxide (food dye; Ruth GmbH & Co.KG, Bochum, Germany) to achieve an opaque white colored appearance which simplified the detection with optical sensors. Titanium dioxide is commonly used in food production [47]. For this cultivation step, ten 500-mL polypropylene containers containing approximately 80 mL of medium were used, in each of which four seedlings were placed for Trial A and five seedlings for Trial B.

### Culture conditions

A polyvinyl chloride foil (PVC system foil; Klarsichtpackung GmbH, Hofheim, Germany) sealed each vessel as a substitution of the lid to provide a fully transparent view while ensuring the aseptic condition of the cultures for both experiments. The cultures were incubated for either 21 days (Trial A) or 16 days (Trial B) for *A. thaliana* and 32 days for *N. tabacum* at 25 °C with a 16 h photoperiod (7 am till 11 pm) and a PPFD (Photosynthetic Photon Flux Density) of 35 to 40 µmol m^−2^ s^−1^, provided by two tubular fluorescent lamps (Philips MASTER TL-D 58W/865). The lab’s bottom-cooling system—provided by water-cooled plastic tubes below the shelf—prevented water condensation due a local shift of dew point (Fig. 2). Room temperature ranged from 19 °C (night) to 25 °C (day) with an average of 22 °C, while the average surface temperature of the cooled cultivation area ranged from 19 °C (night) to 24 °C (day) with an average of 21 °C.

### Optical properties of culture vessel

Spectral transmittance was measured with an UV/VIS/NIR spectrometer (PerkinElmer Lambda 1050) equipped with 150 mm indium gallium arsenide (InGaAs) integrating sphere in a 1 nm wavelength interval from 250 to 2500 nm and with a FT-IR Spectrometer (PerkinElmer Spectrum Two) in a 3.75 nm wavelength interval from 2500 to 15,000 nm. Three independent replicates were measured for transmittance curves (Fig. 5). Additionally Haze index was measured with an UV/VIS/NIR spectrometer (PerkinElmer Lambda 1050) in 5 nm intervals and in a wavelength interval from 380 to 780 nm, according to standard test method ASTM D1003 [18]. Thus, the Haze index was calculated by the following equation:

1 $$\mathrm{Haze\, index }\left[\mathrm{\%}\right]= \left(\frac{\mathrm{Diffuse\, transmittance}}{\mathrm{Total\, transmittance}}-\mathrm{Rel}.\ \mathrm{scattered\, tranmisstance\, by\, the\, system}\right)\times 100$$

### Development of the automated phenotyping system

#### Environmental conditions of the application area

Plant in vitro cultures are usually cultivated in multi-layered shelf systems equipped with tubular fluorescent lamps (TFL) as a light source with a photoperiod of 16/8 h in a temperature-controlled culture room (Fig. 2). Plant in vitro culture techniques are characterized among others by the potential of cultivating high numbers of plantlets at minimum space—up to 50 culture vessels can be placed at a cultivation area of ~ 0.6 m^2^ (1000 mm × 600 mm) containing multiple explants. The distances between the different levels of the multi-layer shelf systems are mainly determined by the heat dissipation of the fluorescent tubes, which limits the available space of potential sensor application to 400 mm between cultivation area and TFL. For the purpose of automated phenotyping of explants cultured under common in vitro conditions, we therefore developed a low-cost multi sensor system at minimum space.

#### Phenotyping platform hardware setup

As backbone of the phenotyping system, a commercially available belt-driven xy-gantry was chosen (ACRO system; OpenBuilds, Zephyrhills, USA), that allows direct control of movement via a G-code sent to the native motion controller. The xy-gantry was specified with an accuracy of 0.1 to 0.2 mm by manufacturer. The dimensions of the xy-gantry were reduced to 1000 mm × 600 mm (X, Y) to match the dimensions of the shelf used in the culture room of Leibniz University Hannover (Fig. 2A). To fulfill the specific demands of monitoring in vitro cultures, several hardware components were added to the gantry. In order to control the height of the multi-sensor detector head (Fig. 2), and in particular to accommodate the variable needs of dynamically monitoring different plant species, we installed an additional screw-driven z-axis (C-Beam Linear Actuator, OpenBuilds, Zephyrhills, USA; modified to a stroke length of 60 mm) and connected it to a motion controller. The linear actuator for the z-axis was specified with an accuracy of 0.05 to 0.1 mm by manufacturer. The cable management was ensured by various 3D-printed parts and common cable chains (GitHub repository [28]). Network connection and power supply of the two single-board computers (Raspberry Pi 4 Model B), controlling either the sensors of the detector head or the serial communication of the G-code to the motion controller, were provided by a router and a Power-over-Ethernet switch (Table 2).

#### Detector head hardware setup

The detector head installed at the z-axis of the system consists of four different sensors (Fig. 2B–F) and diverse LEDs for the illumination (Fig. 2G), including a laser distance sensor (Fig. 2C), a low cost RGB camera (Fig. 2D), a micro spectrometer breakout board (Fig. 2E) and a thermal camera board (Fig. 2F).

**Fig. 14:**
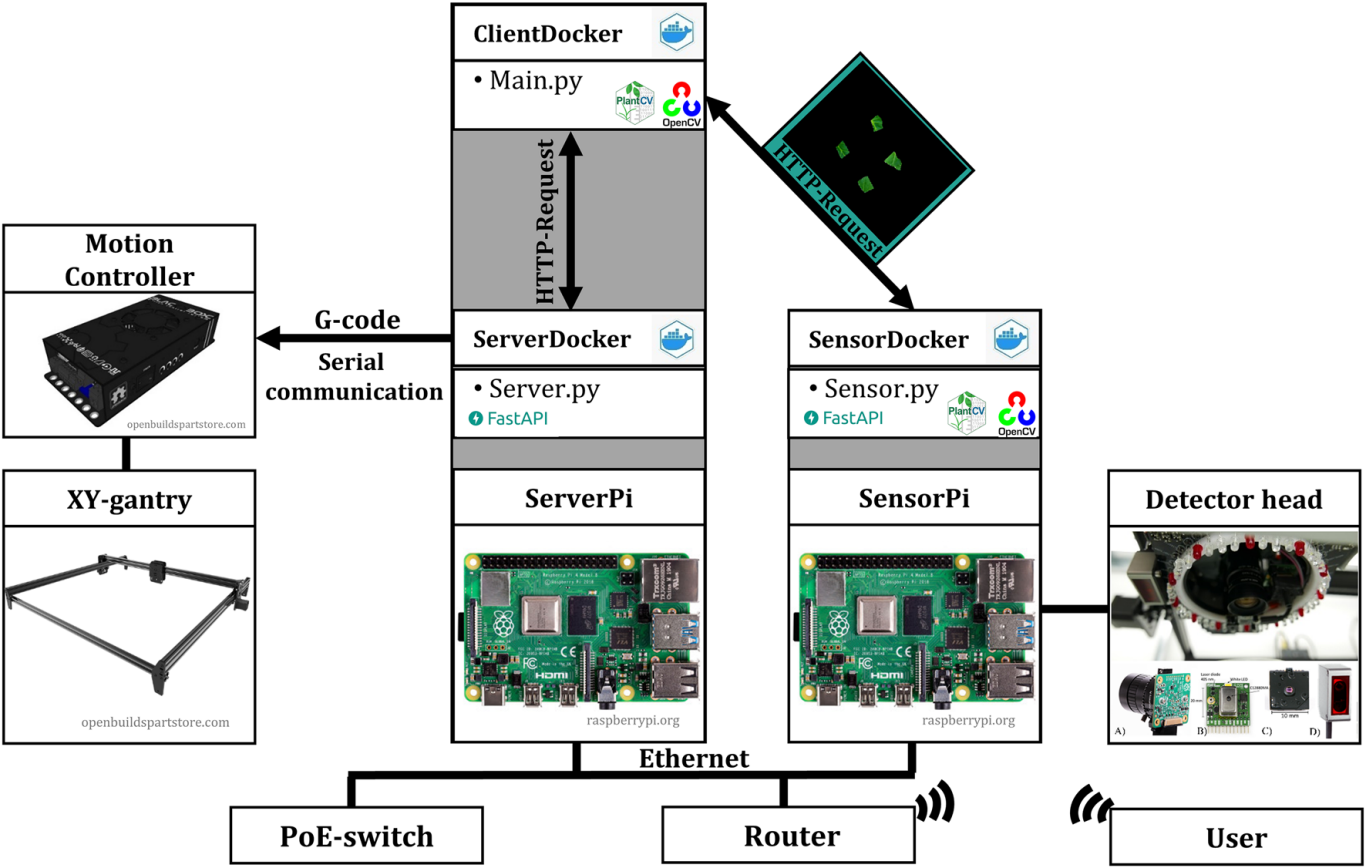
Software design and network communication of the phenotyping system components. Two Raspberry Pis hosting Docker containers executing scripts for the two main tasks of motion control and sensor control and providing the access over HTTP request via the Python framework FastAPI by the main script (semi-autonomous mode/local communication) or the user (reliant mode/ wireless communication). Gray areas represent physically co-located software elements, arrows indicate the direction of data transfer, and black lines mark physically connected hardware components

The laser distance sensor (OD-Mini OB1-B100, Sick AG, Waldkirch, Germany) used in this setup was specified by the manufacturer with a power consumption of < 1.92 W, laser emission wavelength of 655 nm, max. output of 390 µW (laser class 1), a measuring range of 50 to 150 mm and a linearity of ± 100 µm as well as spot size of 700 µm × 600 µm at a measuring distance of 100 mm. The analog output of the laser distance sensor (10 V) was connected via a small voltage divider circuit to a high precision 16-bit A/D-converter (ADS 1115), which communicated via Inter-Integral Circuit (I^2^C) with a microcontroller board (Wemos D1 Mini). The A/D-converter gain was set to 2/3 to read a voltage range of ± 6.144 V and therefore, cover the analog output range of 0 to 5 V. Each distance measurement consisted of a up to ten single readouts and averaging (excluding default sensor values), to achieve a robust and low noise measurement. The microcontroller was powered and read out via USB by the Raspberry Pi of the detector head (Fig. 14: SensorPi).

The 12.3-megapixel RGB camera (Raspberry Pi Camera HQ, Raspberry Pi Foundation, Cambridge, UK) was installed in the center of the ring light PCB to capture top-down images of the in vitro culture vessels (Fig. 2B). The device was electrically connected to a Raspberry Pi via CSI (Camera Serial Interface). The RGB camera was equipped with a 6 mm fixed focal length low-distortion lens (Table 2: Edmund Optics: 6 mm wide angle lens, f/1.2, high resolution = 120 lp/mm, low distortion < 0.5%) to achieve a field of view (FOV) of > 100 mm × 100 mm at a minimum working distance (MWD) of ~ 100 mm, mainly determined by the height of the culture vessels used (500 mL transparent polypropylene containers with a height of 104 mm). Images of in vitro cultures were captured with the following camera parameters: resolution = 4054 px × 3040 px, shutter speed = 2000 ms, iso = 100, autowhite-balance = off and a fixed gain of 3.3, 1.5 (red, blue).

The micro spectrometer board (micro spectrometer and Breakout Board v2, GroupGets, Reno, USA) allows an easy application of the ultra-compact Hamamatsu CMOS image sensor (C12880MA, Hamamatsu Photonics K.K., Hamamatsu, Japan), which has 288 channels with a spectral range of 340 to 850 nm and a spectral resolution of 15 nm. The sensor’s pixel index was converted to wavelength with the device-specific factory calibration coefficients and resulting wavelengths were round to integers. The micro spectrometer board was powered and readout by a microcontroller (Wemos D1 Mini) connected via USB to the Raspberry Pi of the detector head (SensorPi). The analog values of the micro spectrometer were digitized by the 10-bit internal A/D converter of the microcontroller. The micro spectrometer was equipped with a 3D printed tubular aperture (⌀ 5 mm), which was used to integrate a long pass filter (Table 2: Edmund Optics: G420; OD > 5; transmission > 90%) and to limit the detection spot size of the spectrometer. Due to the limited signal, an integration time of 300 ms was specified for fluorescence detection. The dielectric long pass filter with a cut-on wavelength of 420 ± 5 nm was used to separate excitation light of the UV-LEDs (Fig. 2G) from the chlorophyll fluorescence signal measured in dark condition (night).

The thermal camera board (PureThermal 2, GroupGets, Reno, USA) was equipped with the FLIR Lepton 3.5 thermal camera (Lepton 3.5, Teledyne FLIR LLC, Wilsonville, USA). This low-cost device is a radiometrically calibrated thermal camera, sensitive to longwave infrared radiation from 8 to 14 µm, with a spatial resolution of 160 px × 120 px, a horizontal field of view (FOV) of 57°, a radiometric accuracy of up to ± 5 °C and a thermal sensitivity of 0.05 °C. Power supply and data readout was ensured by a USB connection to one of the Raspberry Pis (SensorPi). The internal flat field calibration (dark current correction with closed shutter) was set to be performed every 90 s.

As an essential requirement of the image acquisition of plant in vitro culture, the illumination of the detector head (Fig. 2G) had to limit total reflection, occurring at the culture media surface and the lid of the culture container, to a minimum. Therefore, the illumination setup mainly included diffuse and non-direct lateral illumination. To enable various illumination options and to provide appropriate signal for the spectral measurements, we designed a ring light printed circuit-board (GitHub repository [28]), which consists of 24 white standard LEDs (ROHM Semiconductor: SLA560WBC7T3), 12 UV standard LEDs with a peak maximum of 375 nm (Nichia: NSPU510CS) and 12 red standard LEDs (Lumex: SSL-LX5093HD) with a peak maximum of 700 nm. LEDs were controlled by a Mosfet circuit connected to one of the microcontrollers (Wemos D1 mini) and powered by eight 20 mA micro constant current power supplies. Additionally, a 24 V diffuse ring light with white LEDs and a color temperature of 6500 K was added as the main illumination source for image acquisition.

### Phenotyping platform software setup

The requirements of the software setup comprised (i) remote and programming language independent access and control, and (ii) automatic and robust data transfer from the phenotyping system sensors over weeks. The software design is mainly based on the Python [22] programming language and includes open-source components like Docker [48], FastAPI [49], OpenCV [23] and PlantCv [24] (Fig. 14). To ensure software reproducibility and flexibility—independent of framework and operating system versions—we decided to containerize the applications with Docker (ServerDocker, SensorDocker and ClientDocker) which contain the Python-based main scripts, according to respective task (sensor control, motion control and the fusion of the two tasks). Network communication between the different containers is ensured by a Python framework “FastAPI,” which allows the execution of Python functions, provides the network addressing and the access of sensor data via HTTP requests of the different physically separated network components, resulting in control of the system via HTTP independently of programming languages. The system specific Python library containing all self-defined functions is accessible at our GitHub repository [28]. The hardware and software setup yielded a portable and standalone system, allowing a semi-automated sensor data acquisition.

### Automated data acquisition

#### Step I: Start of the system

Step I included the start of the system and the determination of culture vessel position (Fig. 3). To run the phenotyping system in an autonomous way over weeks of monitoring, the positions of the to be monitored culture containers as a single user input had to be initially set in the Python main script. Alternatively, a vessel detection algorithm based on Circle Hough Transform implemented in our Python library can be used—if some input constants are adjusted to the respective imaging situation. To run the monitoring experiment the started ClientDocker executed the main Python script (Main.py) and thus the library is included with all necessary functions and system constants.

#### Step II: Data structure and capture initial images for determination of plant positions

Once the system is started, the output directory (256 GB USB drive connected to ServerPi) is checked for already existing experiments, then a new experiment folder and subfolders for each culture vessel are created. The culture vessel positions are sequentially approached, capturing initial images and directly determining plant positions of the four largest objects, found by image color space transformation in HSV (hue, saturation and value) and thresholding of the hue channel with Otsu’s method [20] (Fig. 3). Plant positions are calculated by deviation of the centroid of the found objects—converted from pixel to mm—and the known position of image midpoint (xy-position of the motion controller).

#### Step III: Time lapse data acquisition

After the initial steps I and II, the actual time lapse data acquisition is continuously looped over the time of the experiment (Fig. 3). In our experiments, RGB image acquisition was performed sequentially for each culture vessel at the midpoint every 4 h. Thermal images were captured simultaneously with the RGB images, with the thermal sensor shifted to the center point in the xy direction. To determine whether additional illumination is required for RGB night shots, the average pixel intensity of an RGB image previously captured without system illumination was calculated. Once the system recognized a night image situation, the estimated plant positions were sequential approached to capture fluorescence spectral information with the micro spectrometer at the centroid of the found objects and UV excitation lights turned on (Fig. 3). After that, consequently the acquisition of depth data with the point-measuring laser distance sensor was obtained via spatial scan by sequential readout of the sensor point measurements while shifting the detector head in xy direction, according to the scan pattern (e.g., 100 mm × 100 mm; with a resolution of 1 mm × 1 mm) with a speed around 0.27 s per point × 10,000 points per vessel (~ 45 min), which limited the measurement of depth data to two culture vessels per 4-h cycle. For the experiments conducted in this paper, a depth measurement for each culture container once per day was ensured.

### Data processing

#### RGB data processing

Classical image processing approaches—applying thresholds to certain color space channels—failed in different previously conducted experiments due to a high variability and diversity in the obtained image data sets, for instance due to changing illumination situations during the day or due to changes in leaf pigment composition (Fig. 4A). To obtain a robust image classification, we therefore trained a pixel-wise random forest classifier with Ilastik [21]. Ilastik is an open-source toolkit offering machine learning (ML) based image processing for pixel and object classification and tracking. 50 random RGB images of the *A. thaliana* dataset were selected and partially labeled pixel-wise in either background or plant pixels. Features selection was limited to a number of 14 features to reduce computation time (Additional file 7). After verification of the classifier, the model was exported and used in the RGB image processing pipeline in Python script.

PlantCv [24] was used to set up the image processing pipeline allowing a uniform batch-processing of hundreds of images. The trained classifier was executed in the headless mode to obtain the segmentation binary image containing only plant pixels (Fig. 4B). Image processing included an automated brightness and contrast adjustment by histogram stretching and a temporary reduction in resolution to reduce computation time from 4054 px × 3040 px to 1014 px × 760 px while using the pixel-wise classifier. After obtaining the binary plant masks, the connected components analysis was carried out, mainly with established PlantCv functions. For single plant analysis, the following parameters could be calculated: projected plant area, perimeter, convex hull area to calculate solidity/compactness and stockiness (data not shown). Additionally, the cumulative projected plant area of all explants could be determined by the sum of non-zero pixels in the segmented binary plant mask.

#### Depth data processing

The level of zero depth was calculated separately for each culture vessel as the mean of the raw sensor data from four quadrilaterals (10 px × 10 px) of each corner of the scan area (100 mm × 100 mm)—where values only derived from the cultivation surface and not from the media or plant. To obtain depth data from raw sensor values of the laser distance sensor, the calibration curve of a reference object for data conversion was used (Additional file 5). Circle Hough Transform [26] was employed to detect the culture medium in the depth data from Day 0 (Fig. 4C). With the radius (r_1_) of the detected circle, a circular binary mask was created with r_new_ = r_1_ − 3 px, which allowed the removal of disturbing edges of the culture medium for further determination of plant height parameters. RANSAC [18]-based plane detection was therefore applied to the edge-removed point cloud to dynamically identify the eventually tilted medium surface (Fig. 4C). Here, the following parameters were set to detect the planes (distance threshold = 1.5, sample size = 3, iterations = 10,000). The obtained RANSAC plane of the medium was subtracted from the processed point cloud, resulting in height correction and segmentation of the plant depth data. Sum of non-zero pixels of segmented depth pixels (Background: 0, plant: 1) allowed the calculation of plant area by depth data.

With the processed depth data, the following parameters were calculated:

- Medium height (mm): Mean of estimated RANSAC plane
- Medium volume (mm^3^): Assuming a circular conical frustum ($V=\frac{1}{3}\pi h$ (r_1_^2^ + r_1_r_2_ + r_2_^2^); h = medium height, r_2_ = 37 mm; radius of the bottom surface of the culture vessel (constant)
- Average canopy height (mm): Mean of the height corrected plant depth data (output)
- Maximum plant height (mm): Mean of the upper 10 percentile of the corrected plant depth data
- Projeceted plant area_depth data_ (mm^2^): Count of non-zero pixels of segmented and height corrected plant depth data

#### Spectral data processing

For spectral data processing, the analysis focused on the fluorescence measurement, since, in contrast to the spectral reflection, the fluorescence spectra were derived almost exclusively from plant tissue. First, the dark current noise was calculated (spectrometer readout at night, with no excitation light on) as the mean of all dark current measurements. From all fluorescence spectra this mean dark spectrum was then subtracted. For a simplified visualization, the region between 400–660 nm was masked. The masked region contained signals of residual light of the culture room and excitation light (UV) due to imperfect blocking properties of the used longwave filter.

#### Thermal data processing

The image situation in the wavelength region of the spectral sensitivity of the thermal camera was challenging due to the optical properties of the culture vessels. A successful and robust implementation of thermal data acquisition allowed the readout of the 14-bit raw grayscale image by the use of Python library *Flirpy*. Thermal data processing included a conversion of 14 bit grayscale values to °C by manufacturer-specified conversion (y_Celsius_ = y_raw_/100 − 273.15).

### Calibration of the “Phenomenon” phenotyping system

#### Xyz-gantry movement calibration

The motion controller of xy-gantry has been set up with manufacturer-specificized GRBL settings for each axis respectively (GitHub repository [28]), that allow the stepper motor motion to be translated into steps in metric units.

#### RGB sensor calibration

A relation between pixel and metric units was established to express the projected plant area in square millimeters by counting pixels of a graph paper image at the average media height of 20 mm (1 mm = 37.7 px).

#### Laser distance sensor calibration

Laser distance raw sensor data were technically calibrated by measuring a staircase shaped reference object. Therefore, z-axis was set to the same value as used in later experiments (z-axis = − 40 mm = detector head height ~ 130 mm). Reference heights were obtained by a caliper for 6 different heights and 119 raw sensor values were used for calibration. Thus, the zero plane for specific sensor Z-height (z-axis_zero_ = 19430) as well as the maximal valid height could be determined (Additional file 5, maximum height <  = 72 mm). The obtained linear regression function determined the metric conversion of raw sensor data in all conducted experiments.

#### Spectrometer detection spot size determination

Two approaches were used to estimate the measuring spot diameter of the micro spectrometer that had to be modified with a 3D-printed aperture tube (GitHub repository [28]) to reduce the size of the measurement spot: a graphical estimation and an experimental determination. Additional file 8 contains a schematic sketch for the graphical estimation of the detection spot size diameter of 23.5 mm. The experimental determination included a sequential spectrometer readout every 1 mm, while linear movement in x-axis over a grid with black background and white squares of decreasing size and a side length ranging from 30 to 21 mm. Spectrometer channel readouts with the highest signal were picked from the array and plotted over the x axis. We assumed that if the detection spot size diameter is smaller than the side length of the square a constant plateau is found in the respective peak. The first square where a sharp maximum was identifiable, or in particular its side length of 23 mm, determined the detection spot size diameter of this approach.

### Validation of the “Phenomenon” phenotyping system

#### Validation of xy and z-axis repositioning accuracy of the “Phenomenon” phenotyping system

Determination of technical repeatability of xy-axis repositioning over time was conducted by measuring the midpoint deviation by RGB images of a reference object with a flat surface and a height of 41 mm over 16 days for a certain timepoint (12 o’clock), under the settings that were used in all experiments. The initial midpoint (Day 0) of the largest found object in Otsu-binarized L-Channel of CIELAB colorspace was set as the reference for calculation of the mean absolute error (MAE) for x- and y-axis (Table 3). The daily measurement procedure included an initial zeroing through limit switches, repositioning and RGB data acquisition. Reference object surface area of 50 mm × 50 mm and founded counts of px were related to convert the midpoint deviation of  the reference object in px to metric units at a height of 41 mm of the reference object (1 mm = 46.7 px).

**Table 3:** Technical repeatability of xy-gantry repositioning via RGB image analysis

Day [d]	MAE_X_ [px]	MAE_X_ [mm]	MAE_Y_ [px]	MAE_Y_ [mm]
1	8	0.17	3	0.06
2	10	0.21	4	0.09
3	7	0.15	2	0.04
4	8	0.17	7	0.15
5	9	0.19	5	0.11
6	9	0.19	4	0.09
7	10	0.21	3	0.06
8	11	0.24	4	0.09
9	11	0.24	6	0.13
10	12	0.26	6	0.13
11	12	0.26	4	0.09
12	14	0.3	4	0.09
13	15	0.32	6	0.13
14	14	0.3	0	0
15	14	0.3	0	0
Total	10.9	0.23	3.9	0.08

Determination of technical repeatability of z-axis over time was conducted by setting five different z-axis values by the motion controller. Each Z step (0 mm, − 6 mm, − 20 mm, − 40 mm, − 50 mm) was approached five times with initial zeroing through limit switches each time. Actual height changes were recorded by the calibrated laser distance values. Linear regression analysis revealed an R^2^ > 0.99, a MAE_Z_ of 0.09 mm and a RMSE of 0.11 mm (Additional file 4).

#### Validation of the RGB image processing pipeline

The performance of the RGB image processing pipeline, in particular the image segmentation part was checked by manual plant pixel labeling with the annotation software “LabelMe” [50]. 18 randomly selected images from the *A. thaliana* Trial A dataset were used with 3 images per time point and including images of 9 different culture vessels. The 18 binary masks from manual segmentation, thus forming the ground truth dataset, were matched against respective binary masks derived from our RGB image processing pipeline. Plant area was calculated by the sum of non-zero pixels in binary images (Background: 0, plant: 1), while for confusion statistics a full comparison between the two data sets were necessary, revealing 221,834,880 pixel pairs where plant pixels reflected the true positive class and background pixels represented the true negative class.

#### Validation of depth data processing

To estimate the quality of the representation of plants in depth images we selected the projected plant area as a basis of comparison between RGB and depth sensor data and corresponding pipelines, respectively. Therefore, we converted projected plant area by the RGB image processing pipeline from px to square millimeters (37.7 px × 37.7 px = 1 mm^2^), which allowed comparison with projected plant area by depth data processing pipeline. Projected plant area from RGB and depth data of 4 culture containers at 12 time points (n = 48) were submitted to a linear regression analysis, assuming the RGB segmentation as the ground truth data.

### Software environment for data acquisition, processing, analysis and visualization

Data acquisition was done mainly with *Python v3.8.8* [22], using in particular the libraries *FastAPI* [49], *OpenCV v3.4.9* [23], *NumPy v1.20.2* [51], *Serial* v.3.4 [52], *Picamera* v.1.13 [53], *Flirpy* v0.3.0 [54] and with *Arduino IDE 1.8.19* [55] with the following libraries: *arduino-microspec* [56], *SerialCommand* [57] and *Adafruit_ADS1015* [58]

RGB Image processing and analysis was conducted with *Python v3.8.8* [22] in the *Jupyter Notebook v6.3.0* [59] environment using the following packages: *PlantCv v3.11.0* [24], *OpenCV v3.4.9* [23], *NumPy v1.20.2* [51], *Matplotlib v3.4.1* [60], *scikit-image v0.18.1* [61] and the Software toolkit *Ilastik v1.3.3* [26] headless integrated in the Python script.

Depth data analysis included subsequent additional *Python* libraries: *Pandas v1.4.2* [62], *Open3D v0.15.1* [25], *Pyvista v0.34.0* [63].

For data visualization, spectral data analysis and statistical analysis, where statistical test assumptions were proofed graphically, we used *R v4.1.2* [64] and the R-packages *dplyr v1.0.8* [65], *ggplot2 v 3.3.5* [66], *kableExtra v1.3.4* [67], *purrr v0.3.4* [68], *readr v2.1.2* [69], *tidyverse v1.3.1* [70], *hyperSpec v0.100.0* [71] and *photobiology* [72].

### Supplementary Information

**Additional file 1.** Time lapse video of shoot regeneration of N. tabacum in vitro. Leaf explants were cultivated at MS medium supplemented 4.44 µM. Shoot development was were monitored over 32 days of cultivation. Images were segmented with a trained classifier. Uncompressed video is available from the corresponding author on reasonable request.

**Additional file 2.** Time lapse video with original images of A. thaliana growth in vitro. 10 days old seedlings were cultivated on modified B5 mediumand monitored for 16 days. Uncompressed video is available from the corresponding author on reasonable request.

**Additional file 3.** Time lapse video with segmented images of A. thaliana growth in vitro. 10 days old seedlings were cultivated on modified B5 mediumand monitored for 16 days. Images were segmented with a trained classifier. Uncompressed video is available from the corresponding author on reasonable request.

**Additional file 4.** Technical repeatability of Z-axis repositioning. Five different z-axis values were set to the motion controller and approached five times with initial zeroing through limit switches each time. Actual height changes were recorded by the calibrated laser distance values.

**Additional file 5.** Calibration of laser distance sensor. Linear regression of raw sensor values of the laser distance sensor. The reference height was determined with a caliper of a staircase-shaped object (RGB and depth image in bottom left corner). The regression line is colored black, while the linear regression extrapolation is drawn dashed. Gray indicates confidence interval limits at α = 0.95. Adj R² denotes the coefficient of determination adjusted according to Yin and Fan [27], while Pslope and Pinter represent p-values of the coefficients for the intercept and slope determined by simple T-test. MAE and RMSE indicate the mean absolute error and the root mean square error of calibration. n = 119.

**Additional file 6.** Technical repeatability of spatial scanning with laser distance sensor over time. Determination of technical repeatability over time was conducted by measuring a reference object with a flat surface and a height of 41 mm once per day over 6 days, under the settings that were used in all experiments. The initial depth measurementof an area of 50 mm × 50 mm was set as the reference for calculation of the mean absolute errorand the root mean square error. The daily measurement procedure included an initial zeroing through limit switches, repositioning and depth data acquisition by spatial scan.

**Additional file 7.** Random forest classification model features for segmentation of A. thaliana Trail A.

**Additional file 8.** Experimental and graphical determination of modified spectrometer detection spot size. Image of the modified spectrometer are shown in upper right corner. A) Experimental determination of spectrometer detection spot size by a sequential spectrometer readout every 1 mm, while linear movement in x-axis over a grid with black background and white squares of decreasing size and a side length ranging from 30 to 21 mm. Spectrometer channel readouts with the highest signal were picked from the array and plotted over the x-axis. We assumed that if the detection spot size diameter is smaller than the side length of the square a constant plateau is found in the respective peak. The first square where a sharp maximum was identifiable, or in particular its side length of 23 mm determined the spot size diameter. B) Graphical estimation by drawing at a 1:1 scale. Graphical determination found a spot size diameter of 23.5 mm.

## Data Availability

The dataset supporting the conclusions of this article (Hard- and Software of “Phenomenon” phenotyping system) are available in an open-access Github repository, https://github.com/halube/Phenomenon. Supporting information contain beside time lapse videos of conducted experiments, mainly “Methods” section supporting figures and tables. The images data sets of the biological experiments are available from the corresponding author on reasonable request.
