# Correction: Low-cost and automated phenotyping system “Phenomenon” for multi-sensor in situ monitoring in plant in vitro culture

**DOI:** 10.1186/s13007-023-01111-0

**Published:** 2023-11-25

**Authors:** Hans Bethge, Traud Winkelmann, Patrick Lüdeke, Thomas Rath

**Affiliations:** 1 Laboratory for Biosystems Engineering, Faculty of Agricultural Sciences and Landscape Architecture, Osnabrück University of Applied Sciences, Oldenburger Landstraße 24, 49090 Osnabrück, Germany; 2 Institute of Horticultural Production Systems, Section of Woody Plant and Propagation Physiology, Leibniz Universität Hannover, Herrenhäuser Str. 2, 30419 Hannover, Germany; 3 Hannover, Germany

**Correction: Plant Methods (2023) 19:42**

10.1186/s13007-023-01018-w

In the original version of the article, the affiliation ‘Institute of Horticultural Production Systems, Section of Woody Plant and Propagation Physiology, Leibniz Universitätt Hannover, Herrenhäuser Str. 2, 30419, Hannover, Germany’ for first author, Hans Bethge was missing. The original article [1] has been corrected.
