# Supplementary material for: Low-cost and automated phenotyping system “Phenomenon” for multi-sensor in situ monitoring in plant in vitro culture
Source: Plant Methods. 2023 May 2;19:42. doi: 10.1186/s13007-023-01018-w (PMC10152611; doi:10.1186/s13007-023-01018-w)

***Additional file 4- Table 1:*** *Technical Repeatability of Z-axis repositioning*

| Specified Z-axis value [mm] | Calibrated laser distance sensor readout [mm] | MAE [mm] |
| --- | --- | --- |
| 0 | 0.000 | 0.000 |
| 0 | -0.041 | -0.0401 |
| 0 | -0.011 | -0.0111 |
| 0 | -0.037 | -0.0373 |
| 0 | -0.034 | -0.0335 |
| -6 | -6.028 | 0.0283 |
| -6 | -6.025 | 0.0246 |
| -6 | -6.021 | 0.0209 |
| -6 | -6.058 | 0.0581 |
| -6 | -6.017 | 0.0171 |
| -20 | -20.078 | 0.0782 |
| -20 | -20.082 | 0.0820 |
| -20 | -20.082 | 0.0820 |
| -20 | -20.097 | 0.0969 |
| -20 | -20.086 | 0.0857 |
| -40 | -40.142 | 0.1416 |
| -40 | -40.138 | 0.1379 |
| -40 | -40.142 | 0.1416 |
| -40 | -40.179 | 0.1788 |
| -40 | -40.183 | 0.1826 |
| -50 | -50.156 | 0.1565 |
| -50 | -50.171 | 0.1714 |
| -50 | -50.171 | 0.1714 |
| -50 | -50.130 | 0.1304 |
| -50 | -50.201 | 0.2012 |
| **Total** |  | **0.0923** |


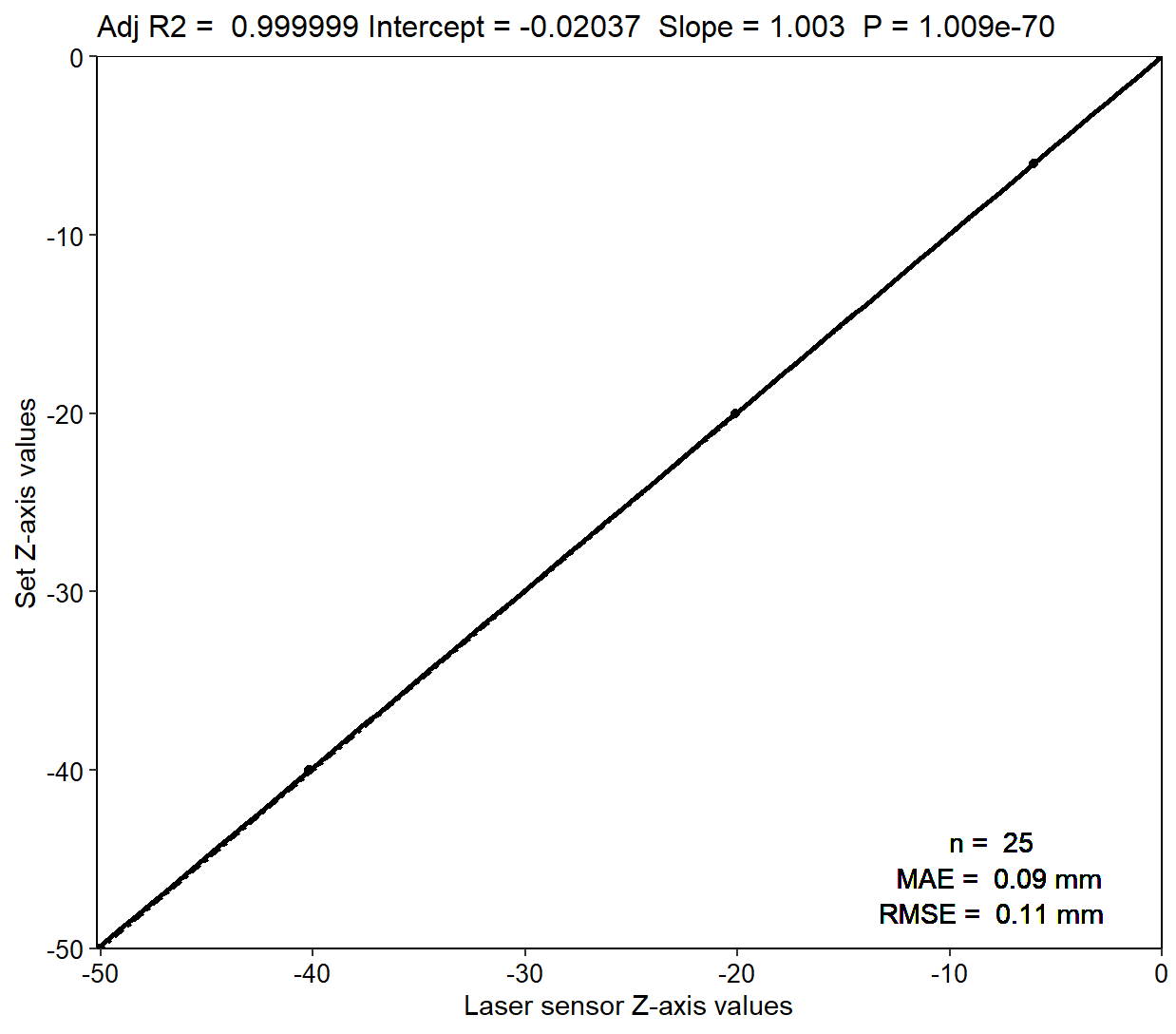

Supplement: Supplementary file 4 — Additional file 4. Technical repeatability of Z-axis repositioning. Five different z-axis values were set to the motion controller and approached five times with initial zeroing through limit switches each time. Actual height changes were recorded by the calibrated laser distance values. [file 13007_2023_1018_MOESM4_ESM.docx]
