# Supplementary figures and images for: Low-cost and automated phenotyping system “Phenomenon” for multi-sensor in situ monitoring in plant in vitro culture
Source: Plant Methods. 2023 May 2;19:42. doi: 10.1186/s13007-023-01018-w (PMC10152611; doi:10.1186/s13007-023-01018-w)

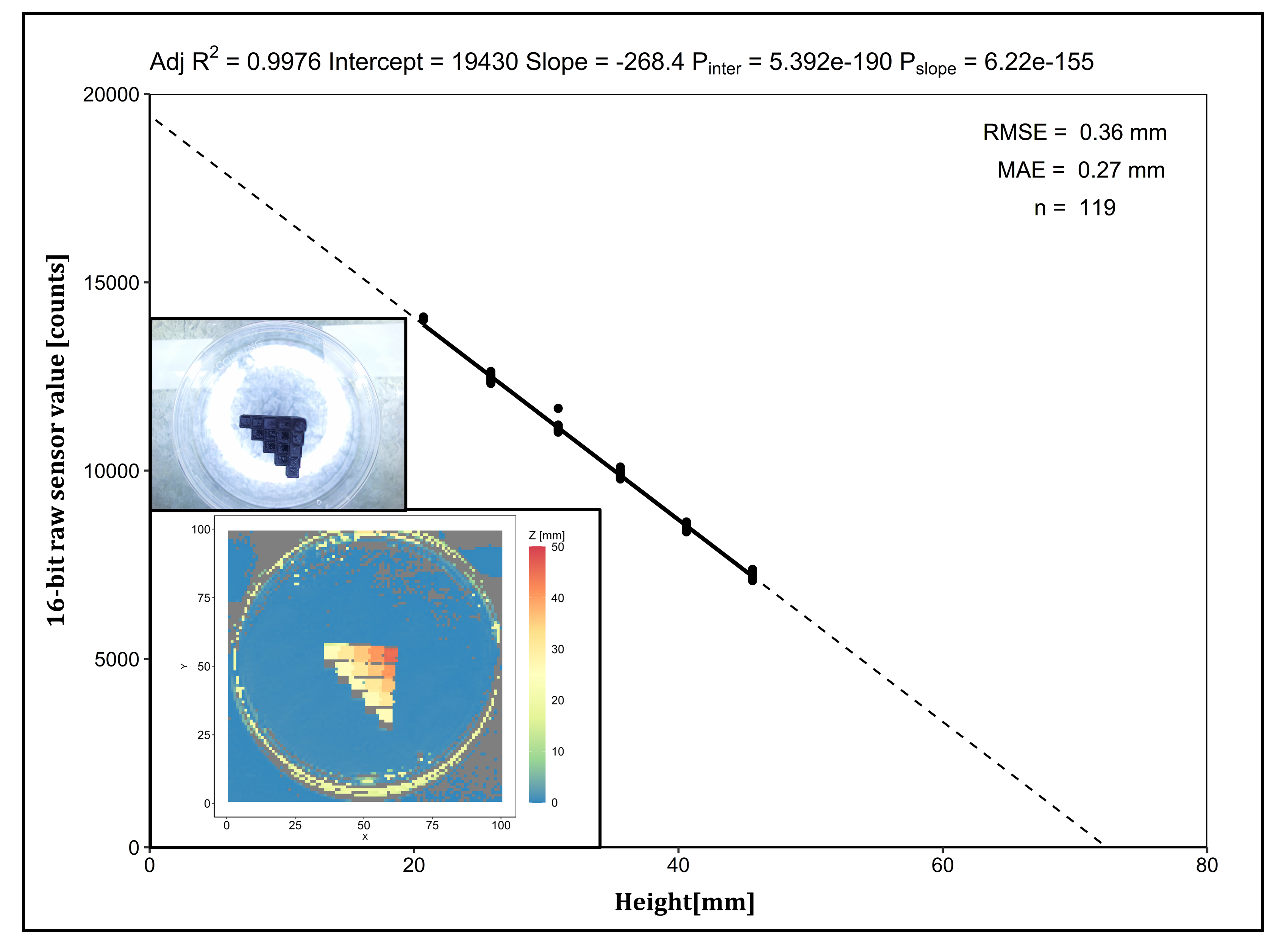

Supplement: Supplementary file 5 — Additional file 5. Calibration of laser distance sensor. Linear regression of raw sensor values of the laser distance sensor. The reference height was determined with a caliper of a staircase-shaped object (RGB and depth image in bottom left corner). The regression line is colored black, while the linear regression extrapolation is drawn dashed. Gray indicates confidence interval limits at α = 0.95. Adj R² denotes the coefficient of determination adjusted according to Yin and Fan [27], while Pslope and Pinter represent p-values of the coefficients for the intercept and slope determined by simple T-test. MAE and RMSE indicate the mean absolute error and the root mean square error of calibration. n = 119. [file 13007_2023_1018_MOESM5_ESM.png]

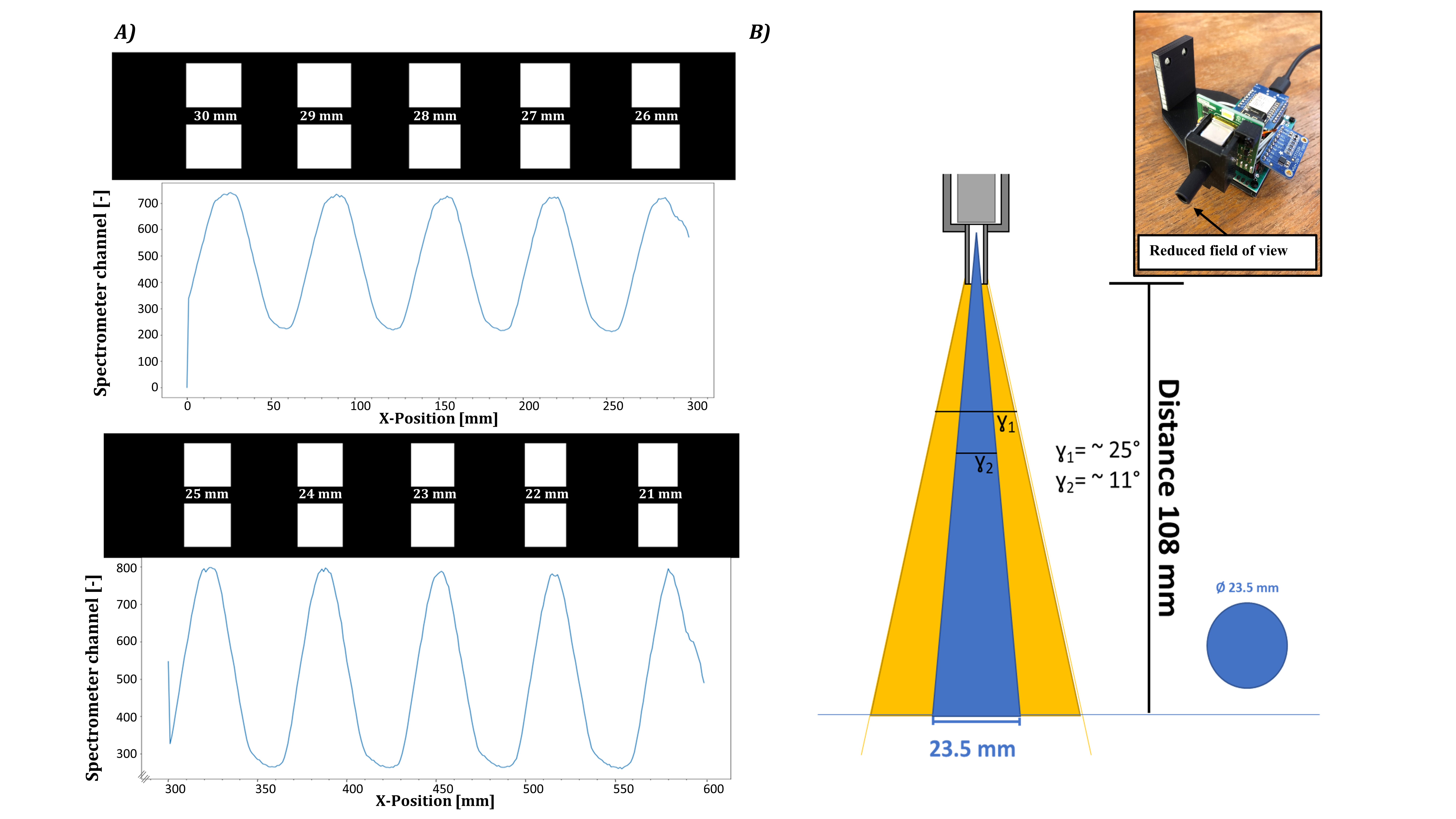

Supplement: Supplementary file 8 — Additional file 8. Experimental and graphical determination of modified spectrometer detection spot size. Image of the modified spectrometer are shown in upper right corner. A) Experimental determination of spectrometer detection spot size by a sequential spectrometer readout every 1 mm, while linear movement in x-axis over a grid with black background and white squares of decreasing size and a side length ranging from 30 to 21 mm. Spectrometer channel readouts with the highest signal were picked from the array and plotted over the x-axis. We assumed that if the detection spot size diameter is smaller than the side length of the square a constant plateau is found in the respective peak. The first square where a sharp maximum was identifiable, or in particular its side length of 23 mm determined the spot size diameter. B) Graphical estimation by drawing at a 1:1 scale. Graphical determination found a spot size diameter of 23.5 mm. [file 13007_2023_1018_MOESM8_ESM.png]
