# Supplementary material for: Low-cost and automated phenotyping system “Phenomenon” for multi-sensor in situ monitoring in plant in vitro culture
Source: Plant Methods. 2023 May 2;19:42. doi: 10.1186/s13007-023-01018-w (PMC10152611; doi:10.1186/s13007-023-01018-w)
Supplement: Supplementary file 6 — Additional file 6. Technical repeatability of spatial scanning with laser distance sensor over time. Determination of technical repeatability over time was conducted by measuring a reference object with a flat surface and a height of 41 mm once per day over 6 days, under the settings that were used in all experiments. The initial depth measurementof an area of 50 mm × 50 mm was set as the reference for calculation of the mean absolute errorand the root mean square error. The daily measurement procedure included an initial zeroing through limit switches, repositioning and depth data acquisition by spatial scan. [file 13007_2023_1018_MOESM6_ESM.docx]

***Additional file 6 - Table 2:*** *Technical Repeatability of spatial scanning with laser distance sensor over time*

| Day [d] | MAE [mm] | RMSE [mm] | Sample number [-] |
| --- | --- | --- | --- |
| 1 | 0.31 | 0.32 | 2500 |
| 2 | 0.49 | 0.51 | 2500 |
| 3 | 0.48 | 0.5 | 2500 |
| 4 | 0.47 | 0.49 | 2500 |
| 5 | 0.37 | 0.4 | 2500 |
| **Total** | **0.42** | **0.44** | **12500** |
